# An unknown component of a selective and mild oxidant: structure and oxidative ability of a double salt-type complex having κ^1^O-coordinated permanganate anions and three- and four-fold coordinated silver cations†

**DOI:** 10.1039/c9ra03230d

**Published:** 2019-09-09

**Authors:** Gréta Bettina Kovács, Nóra V. May, Petra Alexandra Bombicz, Szilvia Klébert, Péter Németh, Alfréd Menyhárd, Gyula Novodárszki, Vladimir Petrusevski, Fernanda Paiva Franguelli, József Magyari, Kende Béres, Imre Miklós Szilágyi, László Kótai

**Affiliations:** Research Centre for Natural Sciences, Hungarian Academy of Sciences Magyar Tudósok krt. 2 Budapest H-1117 Hungary kotai.laszlo@ttk.mta.hu; Department of Inorganic and Analytical Chemistry, Budapest University of Technology and Economics Műegyetem Rakpart 3 Budapest H-1111 Hungary; Department of Physical Chemistry and Materials Science, Budapest University of Technology and Economics Budapest Hungary; Faculty of Natural Sciences and Mathematics, Ss. Cyril and Methodius University Skopje Republic of Macedonia; Department of Chemistry, Biochemistry and Environmental Protection, Faculty of Sciences, University of Novi Sad Trg Dositeja Obradovića 3 Novi Sad 21000 Serbia; Deuton-X Ltd. Selmeci u. 89 Érd H-2030 Hungary

## Abstract

Compounds containing redox active permanganate anions and complexed silver cations with reducing pyridine ligands are used not only as selective and mild oxidants in organic chemistry but as precursors for nanocatalyst synthesis in low-temperature solid-phase quasi-intramolecular redox reactions. Here we show a novel compound (4Agpy_2_MnO_4_·Agpy_4_MnO_4_) that has unique structural features including (1) four coordinated and one non-coordinated permanganate anion, (2) κ^1^O-permanganate coordinated Ag, (3) chain-like [Ag(py)_2_]^+^ units, (4) non-coordinated ionic permanganate ions and an [Ag(py)_4_]^+^ tetrahedra as well as (5) unsymmetrical hydrogen bonds between pyridine α-CHs and a permanganate oxygen. As a result of the oxidizing permanganate anion and reducing pyridine ligand, a highly exothermic reaction occurs at 85 °C. If the decomposition heat is absorbed by alumina or oxidation-resistant organic solvents (the solvent absorbs the heat to evaporate), the decomposition reaction proceeds smoothly and safely. During heating of the solid material, pyridine is partly oxidized into carbon dioxide and water; the solid phase decomposition end product contains mainly metallic Ag, Mn_3_O_4_ and some encapsulated carbon dioxide. Surprisingly, the enigmatic carbon-dioxide is an intercalated gas instead of the expected chemisorbed carbonate form. The title compound is proved to be a mild and efficient oxidant toward benzyl alcohols with an almost quantitative yield of benzaldehydes.

## Abstract 2

A compound having redox-active permanganate and complexed silver ions with reducing pyridine ligands is used as a mild organic and as a precursor for nanocatalyst synthesis in a low-temperature solid-phase quasi-intramolecular redox reaction.

## Graphical abstract

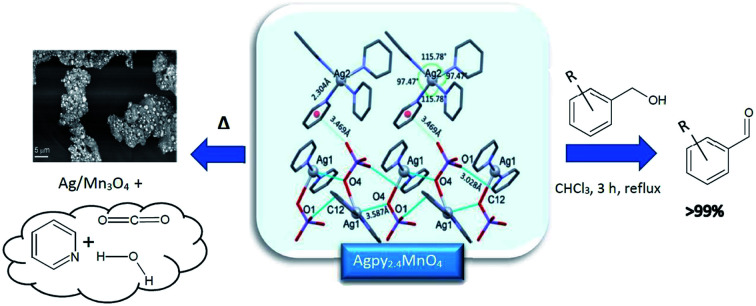

## Introduction

As a useful mild oxidant in organic chemistry, a ‘compound’ prepared by Firouzabadi and reported as ‘[Ag(py)_2_]MnO_4_’ (Firouzabadi's compound, FC) has been known for a long time.^1^ According to literature data, it is an important reagent in a range of oxidation reactions including the preparation of various oxocompounds and sulfones.^1–5^ It can be used as a catalyst for the CN coupling reactions of aromatic hydrocarbons and primary amines including the synthesis of the pharmaceutically important quinazoline heterocycles^3^ such as gefitinib, the drug used for treating breast and lung cancers.^5^ Furthermore, FC decomposes into Ag/manganese oxide particles, which are candidates for catalyzing the selective oxidation of 3-picoline into niacin, the key compound of vitamin B3 synthesis.^6^ The successful application of FC in organic oxidation reactions requires detailed knowledge about the structure, properties as well as the catalytically active chemical sites and the reactivity of the compound. However, the chemical identity of FC is questionable^7^ as its structural data are missing. According to Sajó *et al.*^8^ it is indeed a ∼1 : 1 mixture of the 4[Ag(py)_2_MnO_4_]·[Ag(py)_4_]MnO_4_ double salt (compound 1) and the [Ag(py)_2_]MnO_4_ (compound 2).^9^ Therefore, the structural characterization and properties identification are imperative for both compound 1 and 2 in order to appreciate the mild and selective oxidation capacity of FC. In this work we study the structural and vibrational spectroscopic characteristics, as well as thermal properties and reactivity with selected organic materials of compound 1 and compare its structure and properties with the known perchlorate analogues (listed in Table 1) and selected pyridinesilver(i) permanganate compounds found during the syntheses of FC.^8^

**Table 1:** Studied compounds

Compound	X = Mn	X = Cl
4[Ag(py)_2_XO_4_]·[Ag(py)_4_]XO_4_	1	1-ClO_4_
[Ag(py)_2_]XO_4_	2	2-ClO_4_
[Ag(py)_4_]XO_4_	3	3-ClO_4_
[Ag(py)_2_]XO_4_·0.5py	4	—
∼1 : 1 mixture of 1 and 2	FC	—
Decomposition intermediate from compound 1 at 300 °C	I-300	—

## Results and discussion

### Syntheses

Compound 1 is prepared from the pyridine solution of AgMnO_4_ by dilution with water until reaching 10% pyridine concentration following the method described in the literature.^8^ The blackish purple block-shaped crystals are stable for a month at room temperature in the dark, but after 1–2 weeks a shiny silvery colour appears on the surface of the crystals, which is not accompanied by bulk compositional change (ESI1†).

### Crystal structure of compound 1

**Fig. 1:**
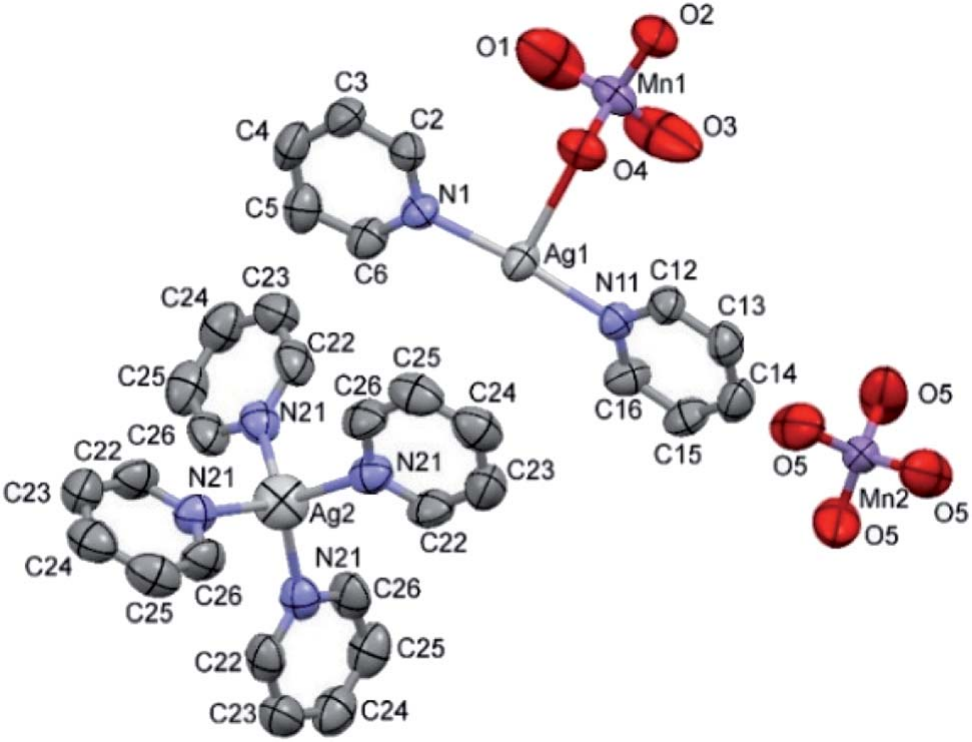
ORTEP presentation of the structure and atomic numbering scheme of compound 1.

The double salt 1 (Fig. 1) crystallizes in the tetragonal crystal system in the space group *I*4̄ (*a* = 21.982(3) and *c* = 7.5974(15) Å, *T* = 293(2) K, *Z* = 2, *D*_calcd_ = 1.885 g cm^−3^). There is one [Ag(py)_2_MnO_4_] and a quarter of [Ag(py)_4_]MnO_4_ in the asymmetric unit. The ratio of [Ag(py)_2_MnO_4_] and [Ag(py)_4_]MnO_4_ is 1 : 4. The Kitaigorodskii packing coefficient is 69.6%,^10^ and there is no residual solvent accessible void in the crystal lattice.

The asymmetric unit contains [Ag(py)_2_MnO_4_] and [Ag(py)_4_]MnO_4_ in the stoichiometric ratio of 1 : 1/4. The displacement ellipsoids are drawn at the 50% probability level.

**Fig. 2:**
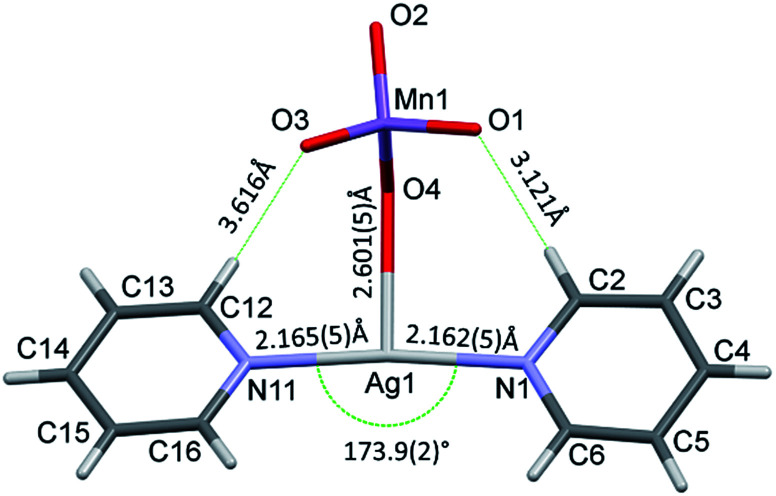
The [Ag(py)_2_MnO_4_] moiety in 1 shows the bond distances and angle around the Ag^+^.

**Fig. 3:**
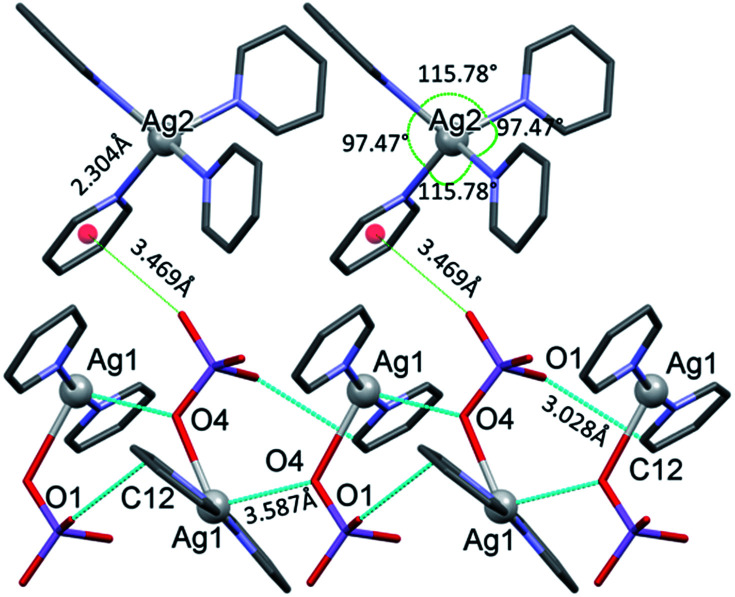
The [Ag(py)_2_MnO_4_] chains along the ‘*c*’ crystallographic axis and their Mn1–O2⋯π interactions (cyan dotted lines) with [Ag(py)_4_] cations in 1.

**Fig. 4:**
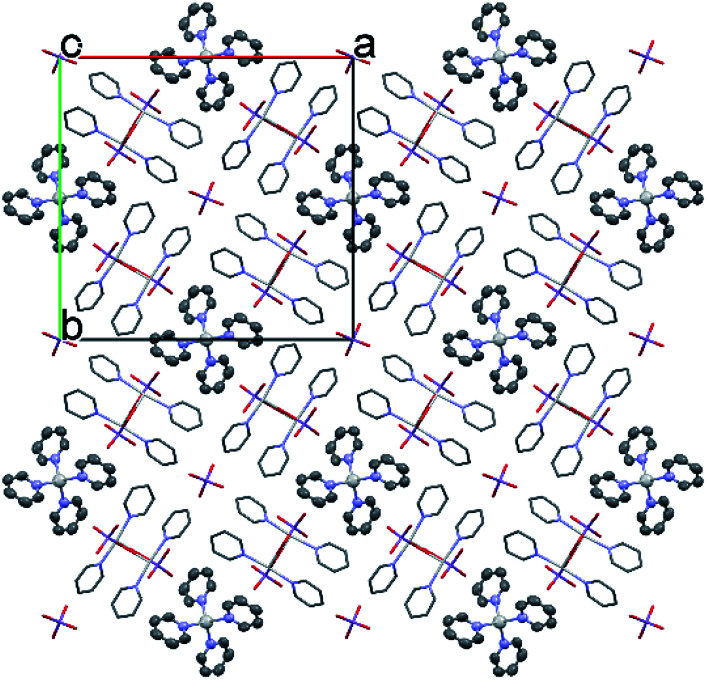
The packing arrangement of 1 [Ag(py)_2_]MnO_4_·[Ag(py)_4_]MnO_4_ viewing from the *c* crystallographic axis.

The conformation of the [Ag(py)_2_MnO_4_] moiety is shown in Fig. 2. The pyridine rings are rotated by an angle of 12.03° respect to each other. The C_α_–H⋯O_permanganate_ interactions are although weak, they contribute to the complex stability. The salt forms chains along the ‘*c*’ crystallographic axis (Fig. 3). These chains are arranged in a framework structure presented in Fig. 4.

The conformation of the [Ag(py)_4_]^+^ is shown in Fig. 3. The Ag^+^ cation is placed on a 4̄ inversion axis. The [Ag(py)_2_MnO_4_] chain and the [Ag(py)_4_]^+^ cations are connected by Mn1–O2⋯π interactions (Fig. 3, where the Mn1–O2⋯Cg(py) distance is 3.469(7) Å and its angle is 161.0(3)°). The framework constructed by the interacting pyridine complexes contains neither classical hydrogen bonds nor π⋯π interaction (Fig. 4).

### Analogue perchlorate compounds

The perchlorate analogue of compound 1 (1-ClO_4_, DITCEO) is isostructural with compound 1, the crystallographic positions of the [Ag(py)_4_]^+^ cations are almost identical in the two crystal lattices (ESI9†). The unit cell volume of 1-ClO_4_ is 0.85% larger than that of compound 1; their cell similarity index,^11^ is 0.00044.

**Fig. 5:**
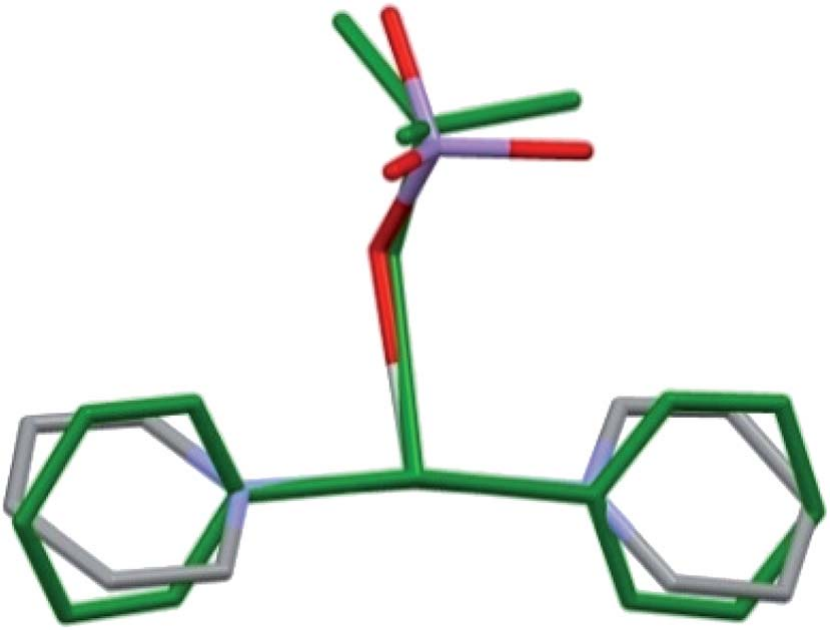
Comparison of the [Ag(py)_2_MnO_4_] moieties from the crystal structures of 1 (coloured by elements) and 4 (green).

The co-crystal 1 has lower stability than compound 1-ClO_4_ (*T*_dec_ = 86 and 95.6 °C,^12–14^ respectively). Compound 3 decomposes fast even at room temperature.^8^ Presence of [Ag(py)_2_]MnO_4_ (2) in its co-crystal (1) with compound 3 stabilizes the tetrapyridinesilver(i) cation in its crystal similarly to the perchlorate analogues of compound 3-ClO_4_ and 2-ClO_4_ in 1-ClO_4_ (*T*_dec_ = 68, 158 and 95.6 °C, respectively^8,14,15^). The conformational arrangement of the coordinated permanganate ([Ag(py)_2_MnO_4_]) containing unit in 1 and 4 (ref. 8) is presented in Fig. 5. Depending on the Ag : py : MnO_4_ stoichiometries of 1 and 4, the [Ag(py)_2_MnO_4_] units have distinct geometries. Superimposing the complexes^17^ the rmsd value is 0.7805 and the max_D_ value is 1.3312 Å.

### Vibrational modes in compound 1

#### Vibrational modes of the coordinated and non-coordinated permanganate anions

Correlation diagrams for MnO_4_^−^ anions at *S*_4_ (non-coordinated permanganate ion) and *C*_1_ (permanganate coordinated to [Ag(py)_2_]^+^ cation) sites of compound 1 lattice are given in ESI2,† the observed IR and Raman frequencies and their assignations are collected in Table 2.

**Table 2:** IR and Raman wavenumbers of permanganate anions in compound 1 at room temperature^a^

Wavenumber, cm^−1^		Assignations
IR	Raman	
826 (w)	826 (vs)	*ν*_1_(MnO), *ν*_s_ (A)
339 (vw)	345 (m)	*ν*_2_(MnO), *δ*_s_ (E)
909, 917 (vs)	887 (w), 902 (w), 913 (w)	*ν*_3_(MnO), *ν*_as_ (F2)
382 (w)	384 (vw)	*ν*_4_(MnO), *δ*_as_ (F2)

a vs-very strong, m-medium, w-weak, vw-very weak.

There are 9 internal modes of the MnO_4_^−^ anions at *S*_4_ sites. The B and E modes of the factor group (f-g) are IR active, whereas all f-g modes (of A, B and E symmetry) are Raman active. E modes are doubly and F modes are triply degenerated. There are 9 internal vibrational modes of the MnO_4_^−^ anions at *C*_1_ sites (permanganates are coordinated to [Ag(py)_2_]^+^ cations). Each mode from the local (site) group splits into 3 components in the factor group. Thus, there are 9 A modes, 9 B modes and 9 E modes (the latter are doubly degenerated) giving rise to 36 vibrational degrees of freedom due to the 4 MnO_4_^−^ anions on general positions (*i.e.* positions with *C*_1_ symmetry). There are 6 external vibrations of the MnO_4_^−^ anions at *S*_4_ sites; 3 and 4 bands are expected in the far IR region, *i.e.* the low-frequency part of the Raman spectra, respectively. The external vibrations of the MnO_4_^−^ anions at *C*_1_ sites result in a total of 24 vibrational degrees of freedom, which is in accordance with the 4 tetrahedral anions on general symmetry positions. The external vibration bands are also expected in the far IR and the low-frequency region of the Raman spectra. No predictions of the band intensities can *a priory* be given.

According to the factor group analysis (unit-cell group analysis), the two types of permanganate ions result in 4 vibrational modes (Table 2). However, the distinctions of permanganate bands belonging to *S*_4_ and *C*_1_ sites are challenging. One would expect a strong doublet due to the symmetric stretch (the breathing mode) in Raman, however, the observed spectrum does not show this expected feature (ESI3†). Three bands appear as a result of splitting of the *ν*_3_(MnO_4_) modes instead of the expected twice three components due to the pronounced distortion of the permanganate moieties at *C*_1_ symmetry.^18*a*,*b*^

### Assigned cation modes of compound 1

Cation vibrations can arise from the hindered Ag translations within the [Ag(py)_2_]^+^ and [Ag(py)_4_]^+^ units. The correlation diagrams for the hindered translations of Ag^+^ cations at *S*_4_ sites and those at *C*_1_ sites are given in ESI2.† A total of 3 vibrational degrees of freedom exist for *S*_4_ and 12 for *C*_1_ sites. All f-g modes are active in Raman scattering, but only B and E modes are IR active. There are 27 internal vibrational modes of the pyridine rings at *C*_1_ sites. Each mode from the local (site) group splits into 3 components in the factor group. There are 27 A, 27 B and 27 E modes (the latter are double degenerated) giving rise to 108 vibrational degrees of freedom. For the three types of pyridine rings, there are 324 vibrational modes of freedom corresponding to internal vibrations of the pyridine molecules. Each mode of external vibrations from the local (site) group splits into 3 components in the factor group. There are 6 A, 6 B and 6 E modes (the latter are double degenerated) giving rise to 24 vibrational degrees of freedom. For the three types of pyridine rings, there are 72 vibrational modes of freedom corresponding to external vibrations of the pyridine molecules. The assignment of the heavily overlapped bands belonging to the pyridine ring is given in ESI2.† The assignments of Ag–N vibrations, arising from the [Ag(py)_2_]^+^ and [Ag(py)_4_]^+^ cations, in comparison with the analogous perchlorate complex (1-ClO_4_)^13^ are given in Table 3. The far-IR spectrum of compound 1 shows two bands at ∼246 and 166 cm^−1^, which are assigned to the symmetric and the antisymmetric *ν*(AgN) modes of the [Ag(py)_2_]^+^ ion. The proposed *ν*_as_(AgN) assignments are in good agreement with the 185 cm^−1^ wavenumber obtained recently from density functional calculations for the species [Ag(py)]^+^.^18*c*^ The *ν*_s_(AgN) mode appearance in the IR spectrum may result from the deviation of the ideal (linear) N–Ag–N angle of [Ag(py)_2_]^+^ ion. Although the N–Ag–N unit slightly deviates from linear (173.9(2)° instead of 180°), this deviation may be sufficient to activate the *ν*_s_(AgN) mode in the IR spectrum.^13^ Since the Ag–N distances in compounds 1 (ESI9†) and 1-ClO_4_ (ref. 12) are practically the same, the difference between the *ν*_s_(Ag–N) band positions in 1 and 1-ClO_4_ suggests that the *ν*_s_(AgN) mode of compound 1 is coupled with *ν*(AgO) modes.

**Table 3:** IR and Raman wavenumbers of complex cations in compound 1 at room temperature^a^

Compound	IR		Raman	
[Ag(py)_2_]^+^ in compound 1	246 (*ν*_s_(AgN))	166 (*ν*_as_(AgN))	247 (*ν*_s_(AgN))	150 (*ν*_as_(AgN))
[Ag(py)_4_]^+^ in compound 1	—	117 (*ν*_as_(AgN))	—	124 (*ν*_as_)AgN
[Ag(py)_2_]^+^ in compound 1-ClO_4_	254 (*ν*_s_(AgN))	164 (*ν*_as_(AgN))	No data	No data
[Ag(py)_4_]^+^ in compound 3	—	122 (*ν*_as_(AgN))^13^	88 (*ν*_s_(AgN))^13^	—

a w-weak, vw-very weak.

A non-separable band system can be seen at frequencies higher than 100 cm^−1^ in the far-IR spectrum of compound 1 (ESI3†), which probably contains the combined bands of the coupled *ν*_as_(AgN) mode of the tetrahedral [Ag(py)_4_]^+^ ion and *ν*(AgO) modes of the O_3_MnO–Ag(py)_2_ units. Although the *T*_d_ symmetry of [Ag(py)_4_]^+^ ion is imperfect, the local symmetry of the AgN_4_ group is sufficiently close to the *T*_d_ symmetry and thus one IR active *ν*_as_(AgN) mode of tetrahedral symmetry is expected for this structure. In compound 1 the fully symmetric *ν*_s_(AgN) mode of A1 symmetry is expected to occur at a lower frequency than our measured Raman range (4000–100 cm^−1^) (the *ν*_s_(AgN) value for 1-ClO_4_ was found at 88 cm^−1^).^13^ The IR frequencies of the tetrahedrally coordinated [Ag(py)_4_]^+^ cations differ from those of the [Ag(py)_2_]^+^, which are consistent with the variations of the Ag–pyridine bond strength and the increasing coordination number in the complexes. The bands at 412 and 418 cm^−1^ for compound 1 (412 and 419 cm^−1^ for compound 1-ClO_4_)^13^ belong to coordinated pyridine modes of the [Ag(py)_2_]^+^ and the [Ag(py)_4_]^+^ cations, respectively. The appearance of single bands for [Ag(py)_2_]XO_4_ (X = Mn (2) and Cl (2-ClO_4_)) at 412 cm^−1^ and for [Ag(py)_4_](XO_4_) (X = Mn (3) and Cl (3-ClO_4_)) at 416 and 419 cm^−1^ confirm these assignations.^8,13^

### UV-spectral studies

The diffuse reflection UV-Vis spectrum of compound 1 contains a band system consisting of pyridine n–π*, Ag^+^-pyridine MLCT and permanganate t_1_–4t_2_, 3t_2_–2e transitions.^18^ The UV-silent counterion containing [Ag(py)_2_]NO_3_ and KMnO_4_ spectra confirm the assignations and multi-complex nature of the UV bands given for compound 1 in Table 4 and ESI4.† The band maxima and their assignations are shown in Table 4.

**Table 4:** Diffuse reflection UV-Vis bands (in nm) of solid 1, [Ag(py)_2_]NO_3_ and KMnO_4_

Compound/band	Compound 1	[Ag(py)_2_]NO_3_	KMnO_4_
Ag–py CT	219.9 mixed band	219.1	—
MnO_4_, ^1^A_1_–^1^T_2_ (t_1_–4t_2_)		—	227.3
MnO_4_, ^1^A_1_–^1^T_2_ (3t_2_–2e)	258.4 mixed band	−252.2	259.0
Pyridine, ^1^A^1^–^1^B_2_ (n → π*)		291.0	—
MnO_4_, (^1^A_1_–^1^T_2_) (t_1_–2e)	521.9	—	499.8, 513.7, 533.4, 562.8
MnO_4_, (^1^A_1_–^1^T_1_) (t_1_–2e)	710.1		720.8

The number of bands belonging to each transition of compound 1 listed in Table 4 can be multiplied due to the presence of two kinds of pyridine containing cations ([Ag(py)_2_]^+^ and [Ag(py)_4_]^+^) and the coordinated/non-coordinated type of permanganate anions. The 219.9 nm band may be assigned as frequencies for combined bands consisting of Ag–py (CT) and MnO_4_ – (^1^A_1_–^1^T_2_ (t_1_–4t_2_)), whereas the 258.4 nm band contains the MnO_4_^−^ (^1^A_1_–^1^T_2_ (3t_2_–2e)) transitions and the components of the pyridine (^1^A_1_–^1^B_2_ (n → π*)) transitions. 219.9 and 258.4 nm bands are close to those found for [Ag(py)_2_]NO_3_ (219.4 and 252.2 nm, respectively) and KMnO_4_ (227.3 and 259 nm, respectively) as well. The main text of the article should appear here with headings as appropriate.

The bands found at 521.9 and 710.1 nm belong to the components of permanganate (^1^A_1_–^1^T_2_)(t_1_–2e) and ^1^A_1_–^1^T_1_ (t_1_–2e) transitions, respectively. The hypsochromic band shift belonging to compound 1 toward ^1^A_1_–^1^T_1_ (t_1_–2e) transition at 521.9 nm of KMnO_4_ might arise from the coordinated nature of permanganates in the lattice of compound 1.

### Thermal decomposition

To initiate the redox reaction between the redox active cationic and anionic parts of compound 1, we heated the material in inert and O_2_-containing atmospheres.^16^ The thermal decomposition process of compound 1 in an inert atmosphere is proved to be a strongly exothermic explosion reaction; a part of the decomposition products practically burns out from the crucible. Therefore, the sample has to be diluted with an inert heat absorbing material (70% alumina). The reaction proceeds with 48.0% (wt) mass loss at 85 °C peak (TG and DTG) temperature (ESI5† and Table 5). The same exothermic characteristic is observed in the experiment performed in air flow (Table 5 and ESI5†), thus the oxygen of the air oxygen is not essential for initiating the decomposition process. The second decomposition step is a slower process in comparison to the first one and occurs between 410 and 500 °C and results in 8.0% (wt) mass loss (DTG peak temperature corresponds to 428 °C).

**Table 5:** Thermal decomposition characteristics of compound 1 in air and inert (He) atmosphere

	Δ*m*, %	Temperatures, °C			
		TG range	DTG peak	DSC range	DSC peak
**Inert atmosphere**					
Step 1	48.0	70–90	85	92.8–101.2	86
Step 2	8.0	410–500	428	—	—

**Under air flow**					
Step 1	48.4	70–110	89	60–110	91
Step 2	7.9	110–220	184	110–210	177
Step 3	2.4	280–750	434	280–500	—
Step 4			744	500–750	744

### The autocatalytic character of the decomposition intermediates

The total mass loss in the decomposition reaction of compound 1 under He suggests the formation of elementary silver and Mn_3_O_4_ (or equivalent amount of Mn_2_O_3_ and MnO) (Δ*m*_theor_ = 55.75%, Δ*m*_found_ = 55.80%). The redox titration of the decomposition product formed at 700 °C with oxalic acid results in close to the 2 : 1 ratio of Mn^III^/Mn^II^ content, and according to the stoichiometry of the compound 1 (Ag : Mn ratio = 1 : 1), the decomposition product consists of ∼60% Ag and ∼40% manganese oxides (γ-Mn_2_O_3_, Mn_3_O_4_ and MnO). Compound 1 contains reducing pyridine ligands, which reduces the silver(i) ions into metallic silver and the permanganate (Mn(vii)) anion into lower valence manganese oxides (MnO_2_, γ-Mn_2_O_3_, Mn_3_O_4_ and MnO). The autocatalytic character of the explosive exothermic decomposition of compound 1 may be attributed to the *in situ* formation of Ag/Mn-oxides (Koerbl's catalyst^19^), which are expected to accelerate the oxidation of such N-heterocycles as pyridine. The complete lack of oxygen evolution (see TG-MS results) and the exothermic character of the decomposition process (ESI5†) even at the initial stage excludes that the decomposition of compound 1 would start with endothermic ligands loss and consecutive oxidation of the liberated pyridine.

**Fig. 6:**
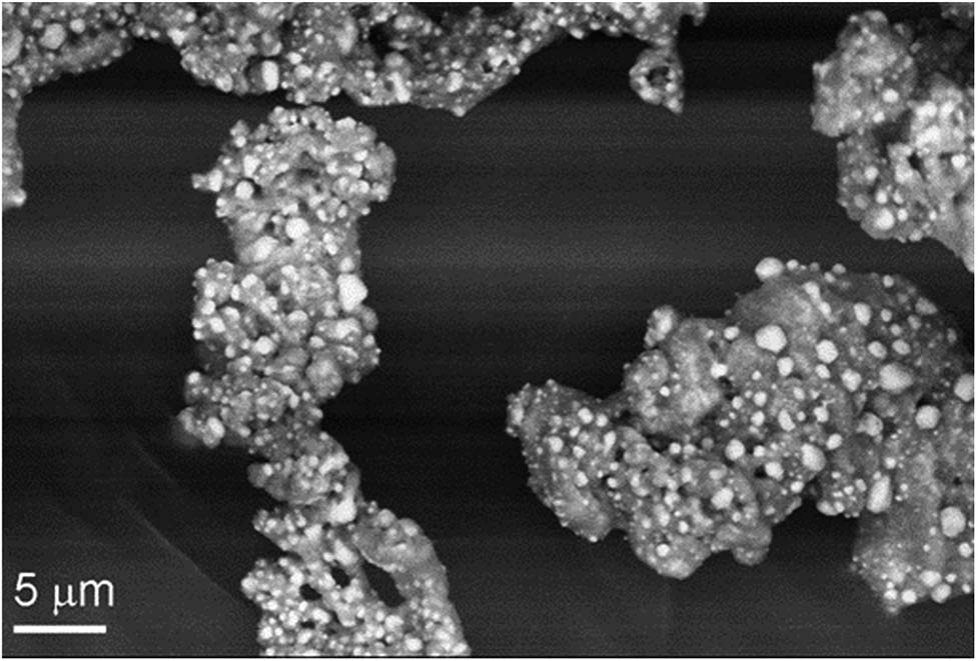
Backscattered SEM image of I-300 (Ag/manganese oxides).

The intermediate formed at 300 °C (I-300) consists of a homogeneous mass of manganese oxides, which is covered by 1–5 μm size crystallites of silver (light grains in Fig. 6). The powder XRD of the intermediate (I-300) confirms the presence of metallic silver, a small amount of MnO and a phase, which could be equally assigned to Mn_3_O_4_ (hausmannite) or γ-Mn_2_O_3_ (ESI6†). These oxides have pseudo-spinel structures with identical lattice constants,^20^ thus PXRD is inappropriate to distinguish between them. Similarly, far-IR studies of I-300 (ESI6†) could not help in these manganese-oxide identifications. The characteristic IR bands of Mn_3_O_4_ and γ-Mn_2_O_3_ are too close to each other;^21^ and IR band shift resulted from the small grain size^22^ and the distribution of Mn^II^ and Mn^III^ cations between the tetrahedral and octahedral sites possibly relating to the ferro/ferrimagnetic ordering of crystals,^23^ makes the IR identification ambiguous.

Since titrimetric chemical analysis of I-300 suggests that the Mn^III^/Mn^II^ ratio is ∼2 : 1, and PXRD does not show γ-Mn_2_O_3_ (bixbyite) contribution, we conclude the main manganese oxide component is Mn_3_O_4_, whereas MnO and γ-Mn_2_O_3_ (in ∼1 : 1 molar ratio) are present in minor amounts. The same PXRD peaks are observed for the product formed at 700 °C and the I-300 (ESI6†) thus the second decomposition step observed at ∼430 °C does not change the chemical identity of the main crystalline phases of I-300. PXRD does not indicate any MnO_2_ contribution, but a weak IR band at 732 cm^−1^ suggests^22*d*^ that it is a minor component of I-300.

**Fig. 7:**
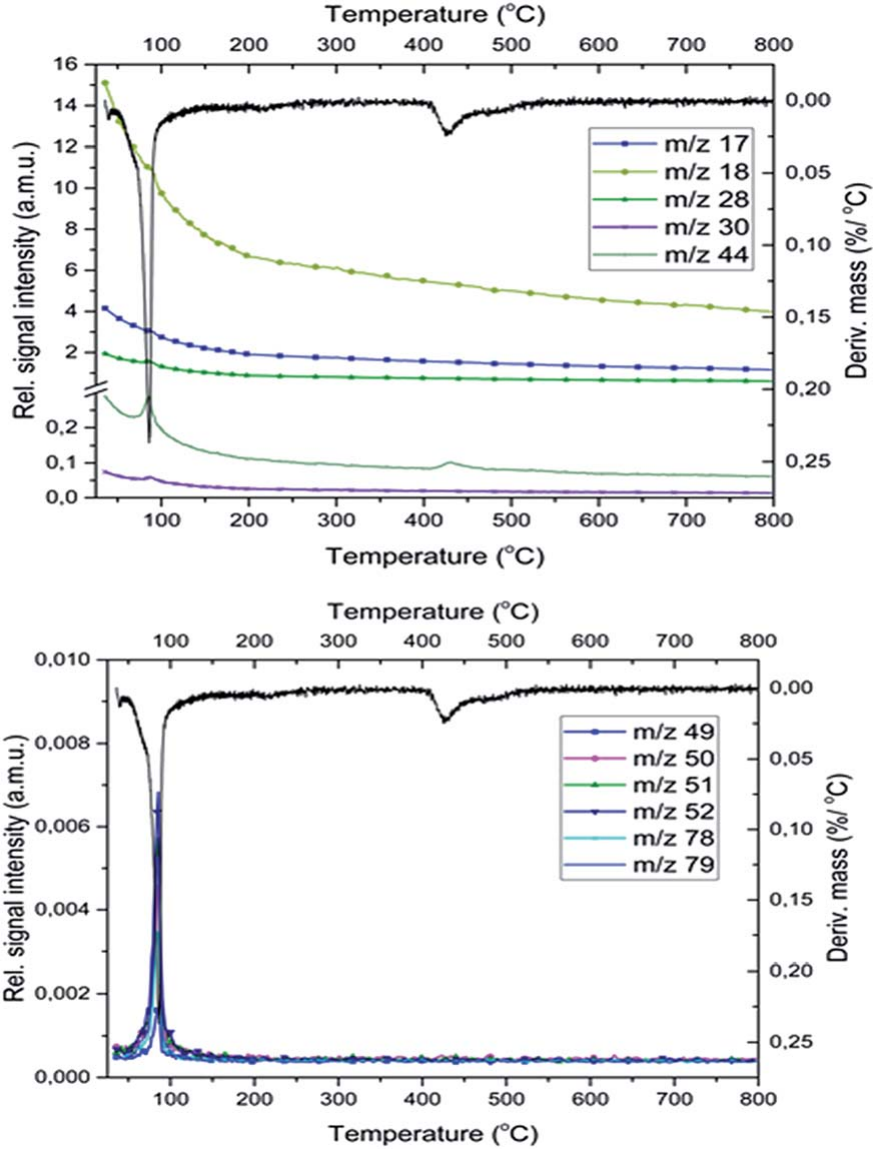
The gaseous products of the redox reaction between the pyridine ligands and permanganate anions in compound 1. The fragment ion intensities belonging to the liberated pyridine during the decomposition of compound 1.

Since the temperatures of the MnO_2_ → Mn_2_O_3_ → Mn_3_O_4_ → MnO decomposition reactions occur at 542, 918 and 1027 °C,^24^ taking into consideration the complete lack of oxygen evolution (Fig. 7), the I-300 phases could unambiguously be formed only from the reduction reactions of permanganate ions. The MnO_2_ IR band disappears on further heating (it is the strongest oxidant among the Mn-oxides present), and the intensity of IR bands belonging to carbon-rich residues in I-300 (aromatic C=C and C=N bonds at 1500–1600 and ∼2300 cm^−1^, respectively) decreases/disappears with the appearance of the oxidative cleavage products of aromatic C=C bonds (C–O–C at ∼1083 cm^−1^) (ESI6†).

### Evolved gas composition and the mechanism of the redox reactions

MS studies on the gas-phase formed during the decomposition of 1 indicate the presence of free pyridine (*m*/*z* = 79, 78, 52), which is liberated together with its oxidation products (CO_2_, H_2_O, NO, and N_2_, *m*/*z* = 44, 18, 30 and 28, respectively) both in the inert and oxygen-containing atmosphere. Based on previous studies^25^ in an inert atmosphere (He) the relative intensities of CO and CO_2_ peaks (*m*/*z* = 28 and 44, respectively) show that the *m*/*z* = 28 signal is a combined signal of CO and N_2_.

The decomposition steps of [Ag(py)_4_]^+^ and [Ag(py)_2_]^+^ cations contemporarily occur in the first decomposition step of compound 1, the ligand losses/oxidations of both cations proceed during a multi-component simultaneous process.

The decomposition temperature of compound 1 (*T*_dec_ = 86 °C) is lower than those compounds that contain the same [Ag(py)_2_]^+^ and [Ag(py)_4_]^+^ cations such as compound 1-ClO_4_ (*T*_dec_ = 95.6 °C),^12^ compound 2-ClO_4_ (*T*_dec_ = 158 °C),^26^ or AgMnO_4_ (*T*_dec_ = 135).^27^ The decomposition of compound 1 is consistent with a solid phase quasi-intramolecular redox reaction and not with a ligand loss/permanganate decomposition followed by consecutive oxidation of the liberated pyridine. The solid phase quasi-intramolecular redox behaviour can be explained by the presence of the weak hydrogen bond interactions between the α-CH hydrogen of pyridine ring in the [Ag(py)_2_]^+^ units and the permanganate ion (Fig. 2) of compound 1. A similar structural feature was observed in compound 4 (hemipyridine solvate of compound 2);^8^ the reaction between the coordinated pyridine ligands and permanganate anions was attributed to the presence of the mentioned N–CH⋯O–Mn hydrogen-bond mediated redox reaction centre.^7*c*^ The evolved heat of the redox reaction between the permanganate anion and pyridine ligands in compound 1 overcompensates the energy demand of the further endothermic ligand loss and initiates the completion of the decomposition process of compound 1 in one main step. In oxygen-containing air flow the decomposition process starts similarly, however, the organic residues of the solid phase are oxidized due to the catalytic activity of the formed Ag and Mn containing redox active intermediate (ESI5†) shown by the exothermic DSC peaks at 177 °C.

### The role of the atmosphere in the decomposition process of compound 1

**Fig. 8:**
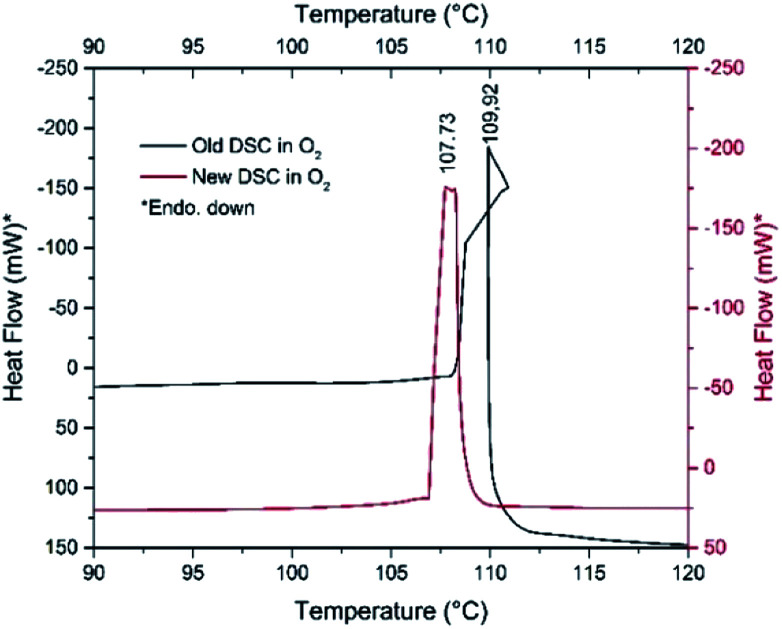
DSC curves of fresh (red line) and one-month-old (black line) samples of compound 1 under O_2_ atmospheres.

The pyridine permanganate = 2.4 molar ratio in compound 1 (CH : MnO_4_ = 12 : 1 → CH/O = 3) suggests that only a part of the pyridine can be oxidized in inert atmosphere by the oxygen content of compound 1, whereas in air, the oxygen content of the atmosphere plays a role in the pyridine oxidation process confirmed by the DSC experiments performed under pure N_2_ and O_2_ (Fig. 8 and ESI7†).

The decomposition heats are −756.94 and −895.02 kJ mol^−1^ under N_2_ and O_2_, respectively. However, sample aging fundamentally influences the decomposition process. The decomposition starting temperature of the fresh material under N_2_ is 107 °C, whereas it is 101 °C for the one-month-old, Koerbl-catalysts containing sample.

The decomposition heat of one-month-old sample under N_2_ slightly increases (−850.35 kJ mol^−1^), which may be attributed to either different reaction products or different distribution of the same products as for the fresh sample. A secondary process may also be found at 129 °C which we explain as the reaction of residual organic content. Under O_2_, the catalyzing effect of decomposition products becomes obvious, the shape of DSC curve and the decomposition heat are strongly altered (Fig. 8). The unusual sigmoid character of the DSC curve is the consequence of the asymmetric heating of the crucible resulted from the non-symmetrical ignition profile of the sample. The decomposition (including the ignition) heat increases significantly, it is −1651.23 kJ mol^−1^ respect to −756.94 kJ mol^−1^ of fresh sample. This increase unambiguously confirms both the role of outer oxygen in the decomposition process and the catalytic effect of the intermediates formed under storage. The appearance of NO and C_4_H_4_ fragments in the TG-MS of compound 1 also suggests that redox reactions of the pyridine rings should proceed similarly to that of compound 2.^8^ Pyridine reduces the permanganate into manganese oxides, which is not accompanied by O_2_ evolution; the permanganate disappears even before its expected decomposition temperature.^7*a*^

The appearance of NO and C_4_H_4_ fragments in the TG-MS of compound 1 also suggests that redox reactions of the pyridine rings should proceed similarly to that of compound 2.^8^ Pyridine reduces the permanganate into manganese oxides, which is not accompanied by O_2_ evolution; the permanganate disappears even before its expected decomposition temperature.^7*a*^

### Enigmatic carbon dioxide in the Ag/manganese oxide matrix

Not only the lack of O_2_ evolution but also the appearance of CO_2_ evolution at 400–430 °C during the decomposition of compound 1 deserves recognition.

This temperature range coincides with the thermal decomposition temperature of MnCO_3_ into MnO and CO_2_, however, there is no indication of MnCO_3_ by PXRD in I-300 and there is no observed reduction of CO_2_ by MnO into CO as it should occur in an inert gas stream:^28^MnCO_3_ = MnO + CO_2_3MnO + CO_2_ = Mn_3_O_4_ + CO

The basic silver carbonate would similarly decompose with CO_2_ evolution in this temperature range,^29^ however, neither PXRD nor IR studies show any silver carbonate^30^ compounds in I-300. Therefore, the *in situ* formed carbonates as sources of CO_2_ evolution could completely be excluded.

The oxidation of carbonaceous materials with MnO_2_ (or partly with Mn_2_O_3_) starts above 300 °C. However, the IR spectrum of I-300 unambiguously shows the presence of intercalated gaseous CO_2_ (2350 cm^−1^)^31^ and its amount decreases significantly during heating up to 700 °C (ESI6†). The formation of this gas can be attributed to the solid-phase redox reaction, during which the formed manganese oxide matrix encloses the locally evolved gas. The high-temperature CO_2_ formation would suggest a strong bond between CO_2_ and the porous manganese oxide sorbents,^32*a*^ which could occur *via* the Mn=O + CO_2_ = Mn(CO_3_) chemisorption reaction. However, the BET surface area does not support strong interaction between the CO_2_ and the Ag/Mn-oxide matrix as the N_2_ and CO_2_ adsorption measurement shows only 6 and 3 m^2^ g^−1^, respectively. The CO_2_ sorption–desorption isotherms of I-300 (ESI8†) also suggest that the sample contains a simple gas inclusion, which is consistent with the gas-impermeable character of the formed mixture.

In the O_2_ atmosphere, three small peaks of CO_2_ formation could be detected during decomposition of compound 1. In addition to the peak of CO_2_ formed at 430 °C in an inert atmosphere, two additional peaks appear at 200 °C and 750 °C due to the formation of carbon dioxide in the aerial oxidation of organic residues.

### The role of the oxygen gas flow on the thermal decomposition process of compound 1

DSC study of compound 1 at 10 °C min^−1^ heating rate under Ar shows that compound 1 has no polymorphic phase transition between −150 °C and the decomposition temperature. The decomposition peak temperatures under Ar or O_2_ atmosphere are found to be almost identical, 101 and 108 °C, respectively (Fig. 8, ESI7†). Under O_2_ gas almost twice more heat is evolved than under Ar, and two very small exothermic peaks (93 and 98 °C) also appear as a result of aerial oxygen during decomposition.

### Organic oxidation reactions

Firouzabadi synthesis results in a mixture of compounds 1 and 2, and the recrystallization of the mixed raw product from benzene gives rise to the formation of pure [Ag(py)_2_]MnO_4_·0.5py (compound 4).^8^ In order to clarify which compound or which component of the reaction mixture prepared and reported by Firouzabadi^1*a*^ is responsible for the mild and useful oxidation effect, the oxidation abilities of compounds 1, 2 and 4 have to be studied on well-selected test materials systematically. As a part of this systematic study, the oxidation ability of compound 1 has been tested in the oxidation reaction of benzyl alcohols (BzOH, 2- and 4-nitro and 2-methoxy-substituted benzyl alcohol) in various organic solvents, at room and reflux temperatures (CHCl_3_-61 °C, CCl_4_-76 °C and benzene-80 °C). The results of oxidation reactions with selected solvents are given in Tables 6 and 7.

**Table 6:** Oxidation of benzyl alcohol (BzOH) with compound 1 at room temperature and solvent reflux temperatures (in the presence of 1.5 fold excess of oxidant, followed by GC-MS)

Solvents	*T*, °C	*t*, min	Reaction products, in %^a^			BzOH conversion
			PhCHO	PhCOOH	PhPh	
CCl_4_	Reflux	30	77.7	20.1	0	Incomplete
CCl_4_	Reflux	120	71.1	28.9	0	Complete
C_6_H_6_	Ambient	180	19.6	3.4	0	Incomplete
C_6_H_6_	Reflux	30	41.9	51.2	4.9	Incomplete
C_6_H_6_	Reflux	120	34.3	60.4	5.3	Complete
C_6_H_6_^b^	Reflux	240	100	0	0	Complete
C_6_H_6_^c^	Reflux	240	61.7	34.2	4.1	Complete

a From GC-MS ion chromatograms. The relative error of measurements was below ±0.4%.

b Using freshly prepared compound 1 without silvery colour.

c In the presence of an artificial silver mirror prepared from diamminesilver(i) chloride and glucose.^33^

**Table 7:** Oxidation of BzOH and some substituted benzyl alcohols (2-NO_2_, 2-MeO and 4-NO_2_) with compound 1 at room and/or reflux temperatures (1.5 fold excess of oxidant, followed by GC-MS) in chloroform

Compounds	Solvent	*T*, °C	*t*, min	Reaction products, in %^a^, PhCHO	
RC_6_H_4_CH_2_OH				RC_6_H_4_CHO	RC_6_H_4_COOH
R = H	CHCl_3_	Reflux	30	89.0	0
R = H	CHCl_3_	Ambient	180	97.3	0
R = 2-MeO	CHCl_3_	Ambient	30	98.9	0
R = 2-NO_2_	CHCl_3_	Ambient	30	100	0
R = 4-NO_2_	CHCl_3_	Ambient	30	100	0
R = 4-NO_2_	CHCl_3_	Reflux	30	100	0

a The relative error of measurements was below ±0.4%.

### Oxidation of benzyl alcohol by 1

Under analogous conditions used by Firouzabadi,^1*a*^ benzaldehyde (PhCHO) and benzoic acid (PhCOOH) are formed together in a PhCHO/PhCOOH = 0.5 ratio. Firouzabadi reported 96% isolated PhCHO yield without by-products,^1*a*^ in contrast, our experiment unambiguously showed that compound 1 could oxidize the PhCHO formed from BzOH further to PhCOOH. A further difference is the appearance of a small amount of diphenyl (PhPh).

**Scheme 1:**
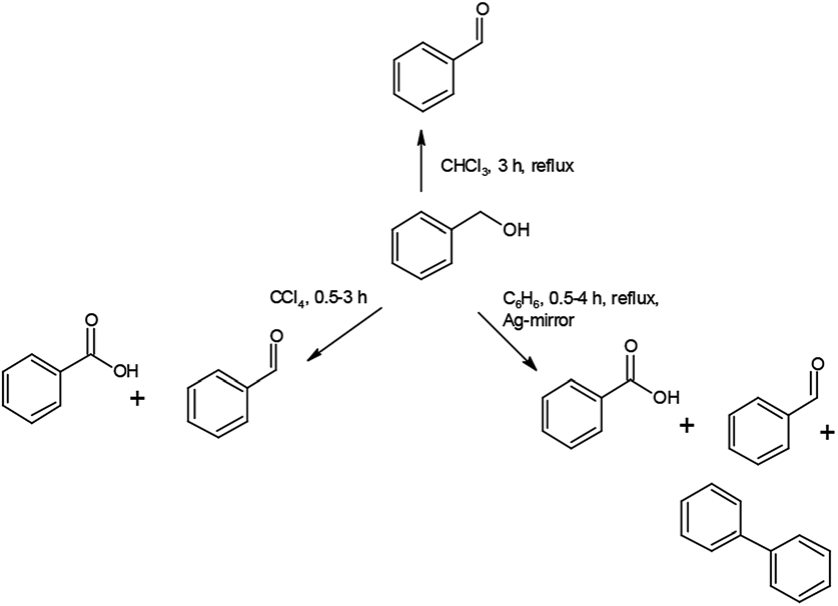
Oxidation reactions of 1 towards benzyl alcohol.

**Scheme 2:**
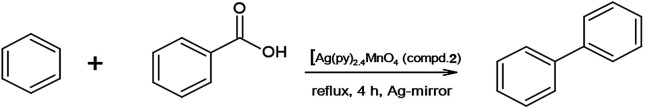
The reaction route to form PhPh.

Increasing reaction time (from 30 to 120 min in benzene as solvent) under reflux slightly influences the distribution of the oxidation products (Table 6). In contrast, the reaction temperature greatly influences the distribution of the products. At room temperature the PhCHO/PhCOOH acid ratio is *ca.* 6 and the BzOH conversion is *ca.* 20%. The appearance of PhPh is observed only at reflux temperature (80 °C) (Schemes 1 and 2).

Since carbon tetrachloride does not result in PhPh formation even at reflux temperature (76 °C) in either 30 or 120 min reaction time, thus the influence of solvents also has to be tested on the formation of PhCOOH and PhPh (Table 6).

Compound 1 is purple when freshly prepared but gets a shiny silvery colour in due time, which is attributed to the formation of a thin surface film of metallic silver, similarly to the AgMnO_4_-sourced silver particles formation.^7*a*^ Silver nucleating centers result in silver mirror formation on the wall of the vessel (both in CCl_4_ and benzene). Since the PhPh formation is observed only in benzene solvent, the decarboxylation of PhCOOH and dimerization of phenyl radicals are improbable, thus benzene is the key factor in the PhPh formation.

The silver catalyzed reaction of benzene with PhCOOH in the presence of a strong oxidant (*e.g.*, persulphate) has already been investigated,^34^ thus the PhPh formation is supposed as a result of the reaction of benzene and PhCOOH with permanganate as oxidant and silver as a catalyst. To confirm this hypothesis, fresh compound 1 (without silvery lustre) was prepared and used it immediately. During 240 min reflux in benzene, it quantitatively yields PhCHO, without diphenyl or benzoic acid formation. Preparing a silver mirror from diamminesilver(i) chloride and glucose as reducing agent,^35^ and using the silver-coated vessel to perform the reaction of BzOH and freshly prepared compound 1 (without silvery lustre), under analogous conditions, a complete BzOH conversion with PhCHO/PhCOOH = ∼2 : 1 ratio and several percent of PhPh formation could be observed. This experiment unambiguously shows the catalytic effect of silver in the PhPh and PhCOOH formation reaction.

Testing of PhCOOH decarboxylation in benzene under reflux for 240 min without any oxidizing agent (*e.g.*, compound 1) fails to form PhPh. Thus, not only the benzene as solvent and metallic silver as a catalyst play key role in the formation of PhPh but also the presence of oxidant is essential to form PhPh.

As it was revealed that the polarity of the solvent and the reaction temperature play an essential role in the conversion of benzyl alcohol and the distribution of the oxidation products, a low-boiling and oxidation-resistant but polar-solvent (CHCl_3_) was also tested in the reaction between compound 1 and various benzyl alcohols (Table 7). Roughly 90% conversion was found in 30 min at reflux temperature, notwithstanding at room temperature, 180 min reaction time gave an almost complete conversion of BzOH into PhCHO without PhCOOH formation.

### Substituted benzyl alcohols oxidation with compound 1

Since the chloroform was found to be the best choice among the tested solvents, with the aim of preparation of the appropriately substituted benzaldehydes, a couple of oxidation reactions were examined. In particular, the 2- and 4-nitrobenzyl alcohols (electron-withdrawing substituents) as well as 2-methoxybenzyl alcohol (electron-donating substituent) substrates were tested. The oxidation reactions were performed in chloroform at room and reflux temperatures (Table 7). The phenyl ring substituents in benzyl alcohol increased the reactivity towards oxidation with compound 1. Independently from the nature and position of the substituents, practically complete conversion of the benzyl alcohols into the appropriate benzaldehyde occurred even at room temperature in 30 min. To examine the effect of temperature, the oxidation reaction of 4-nitrobenzyl alcohol was tested at reflux temperature as well, but the oxidation of the formed 4-nitrobenzaldehyde did not occur at all. These preliminary results confirmed the oxidation ability of compound 1. However, similar structural and reactivity studies of compound 2 are necessary in order to use FC for preparing commercial pharmaceuticals.

## Experimental

In the synthesis and analytical experiments analytical grade pyridine, silver(i) nitrate, 40% aq. NaMnO_4_ and solid KMnO_4_, twice distilled water, hydrochloric acid (37%) and nitric acid (67%) (Deuton-X Ltd) were used. All experiments with pyridine ligand containing silver permanganate and perchlorate complexes have to be performed very carefully due to the existing a possible hazard of explosion. The procedure to prepare the compound 1 can be safely, without risk of explosion, performed in the following way: freshly prepared wet silver(i) permanganate (2.27 g, precipitated in the reaction of saturated aq. AgNO_3_ and 40% aq. NaMnO_4_ solutions, at 1 : 1 Ag : MnO_4_ ratio, with washing the precipitate with copious amounts of cold water) was dissolved in pure pyridine, then the saturated purple solution formed was immediately diluted with water to reach a pyridine content of 10%. A precipitate was immediately formed which proved to be pure 1.^8^ Using wet AgMnO_4_ is essential, because old-samples of AgMnO_4_ can ignite the pure pyridine due to the catalytic effect of the silver manganese oxides formed on the surface of the old and dry samples.

The digestion of samples for ICP measurements was done in the 1 : 1 mixture of 67% nitric acid and 37% hydrochloric acid. The organic (benzyl alcohol, *o*- and *p*-nitrobenzyl alcohol and *o*-methoxybenzyl alcohol) were reagent grade chemicals (Deuton-X Ltd). [Ag(py)_2_]NO_3_ was prepared according to the method given by Jörgenson.^35^

The organic oxidation reactions have been performed with 0.01 mol of benzyl alcohol derivative dissolved in the appropriate organic solvent (CHCl_3_, CCl_4_ or benzene) and 1.5-fold molar excess of compound 1. The mixture was stirred at room temperature for 30 or 120 min, and another portion of the reaction mixture was refluxed (CHCl_3_-61 °C, CCl_4_-76 °C and C_6_H_6_-80 °C) for 30 or 120 min. The conversion was followed by GC-MS ion intensities until reaching the complete conversion. The partial conversion data was calculated from ion-chromatogram intensities which define a rough estimation of molar conversions. The calibration was performed using 2,4-dinitrophenylhydrazones.

Manganese(iii) (Mn_2_O_3_ and Mn_3_O_4_) content of the thermal decomposition products were determined by reacting the mixtures containing these materials with oxalic acid in the presence of 20% sulfuric acid, with 2 h boiling then the oxalic acid excess was titrated back with 0.002 M potassium permanganate solution.

The Ag and Mn content of compound 1 were determined by atomic emission spectroscopy using a Spectro Genesis ICP-OES (SPECTRO Analytical Instruments GmbH, Kleve, Germany) simultaneous spectrometer with axial plasma observation. The multielement standard solution for ICP (Merck Chemicals GmbH, Darmstadt, Germany) was used for calibration. The carbon, hydrogen and nitrogen content were measured by elemental analysis (Fisons model CHN 1018S). The phase purity of compound 1 was checked by powder X-ray diffractometry. X-ray powder diffraction measurements were performed using a Philips PW-1050 Bragg–Brentano parafocusing goniometer equipped with a Cu tube operated at 40 kV and 35 mA power. A secondary beam graphite monochromator and a proportional counter were also equipped. Scans were recorded in step mode. Full profile fitting techniques were used for the evaluation of the diffraction patterns.

The FT-IR spectrum of compound 1 was recorded in the attenuated total reflection (ATR) mode on a Bruker Tensor 27 Platinum ATR FT-IR spectrometer (2 cm^−1^ resolution) between 4000 and 400 cm^−1^. The far-IR measurement was performed on a BioRad-Digilab FTS-30-FIR spectrometer for the 400–40 cm^−1^ range in polyethylene pellet.

The Raman measurement was performed using Horiba Jobin-Yvon LabRAM-type microspectrometer with external 532 nm Nd-YAG laser source (∼40 mW) and Olympus BX-40 optical microscope. The laser beam was focused by an objective (10×) and a D1 intensity filter decreased the laser power to 10% to avoid thermal degradation. The confocal hole of 1000 μm and 1800 groove mm^−1^ grating monochromator were also used in a confocal system and for light dispersion, respectively. The spectral range of 100–4000 cm^−1^ was detected with 3 cm^−1^ resolution. Each spectrum was collected at 240 s per point.

Diffuse reflectance spectrum in the UV-Vis region (200–800 nm) was detected at ambient conditions by a Jasco V-670 UV-Vis spectrophotometer equipped with NV-470 type integrating sphere using the official BaSO_4_ standard as a reference.

Thermal data were collected using a TA Instruments SDT Q600 thermal analyzer coupled to a Hiden Analytical HPR-20/QIC mass spectrometer. The decomposition was followed between room temperature and 800 °C at 2 °C min^−1^ heating rate in He and air as carrier gas (flow rate = 50 cm^3^ min^−1^). Alumina crucible and an empty alumina crucible were used as a sample holder and as a reference, respectively. Sample mass was ∼14 mg, 30% compound 1 with 70% (wt) calcined alumina. Selected ions between *m*/*z* = 1–120 were monitored in Multiple Ion Detection Mode (MID).

The non-isothermal DSC curve between −150 and 170 °C was recorded using a PerkinElmer DSC 7 apparatus. Sample mass was 4 mg and measured at 10 °C min^−1^ heating rate under continuous nitrogen flow (20 cm^3^ min^−1^) in an unsealed aluminum pan. The measurement was also performed under O_2_ flow between −50 and 170 °C.

Scanning Electron Microscopy (SEM) measurements were performed using a Zeiss EVO40 microscope operating at 20 kV. The single crystals of 4[Ag(py)_2_MnO_4_]·[Ag(py)_4_]MnO_4_ (1) were grown from the pyridine solution of AgMnO_4_ by adding 10-fold amount excess of water and leaving the solution to crystallize at room temperature. The diffraction pattern of the blackish purple, block type single crystal with the size of 0.25 × 0.25 × 0.20 mm was measured. Cell parameters were determined by least-squares using 30 439 (6.78 ≤ *θ* ≤ 25.28°) reflections. Intensity data were collected on a Rigaku RAxis Rapid II diffractometer (monochromator; Mo-Kα radiation, *λ* = 0.71073 Å) at 293(2) K in the range 3.389 ≤ *θ* ≤ 25.244.^36^ A total of 21 264 reflections were collected of which 1912 were unique [*R*(int) = 0.0301, *R*(*σ*) = 0.0188]; intensities of 1805 reflections were greater than 2*σ*(*I*). Completeness to *θ* = 0.994. The crystal contains two Ag complex cations with pyridine molecules as ligands and two permanganate anions. The ratio of the two complexes is 1 : 4 in the double salt. The lattice has high *I*4̄ symmetry. It results in low data to parameter ratio. In case of one tetrahedral cation and one tetrahedral anion there is only one-fourth of the molecule in the asymmetric unit.

Numerical absorption correction was applied to the data (the minimum and maximum transmission factors were 0.8567 and 0.9965).

The structure was solved by direct methods.^36^ Anisotropic full-matrix least-squares refinement^36^ on *F*^2^ for all non-hydrogen atoms yielded *R*1 = 0.0208 and w*R*_2_ = 0.0440 for 1332 [*I* > 2*σ*(*I*)] and *R*1 = 0.0235 and w*R*_2_ = 0.0445 for all (1912) intensity data, (number of parameters = 230, goodness-of-fit = 1.032, the maximum and mean shift/esd is 0.000 and 0.000). The absolute structure parameter is 0.015(9) (Friedel coverage: 0.821, Friedel fraction max.: 0.998, Friedel fraction full: 0.998). The maximum and minimum residual electron density in the final difference map was 0.386 and −0.274 e Å^−3^. The weighting scheme applied was *w* = 1/[*σ*^2^(*F*_o_^2^) + (0.02940.0000*P*)^2^ + 0.0000*P*], where *P* = (*F*_o_^2^ + 2*F*_c_^2^)/3.

Hydrogen atomic positions were calculated from assumed geometries. Hydrogen atoms were included in structure factor calculations, but they were not refined. The isotropic displacement parameters of the hydrogen atoms were approximated from the *U*(eq.) value of the atom they were bonded to.

The liquid products were analyzed using a GC-MS (Shimadzu QP2010, He as the carrier gas) equipped with an RXi-5SIL MS capillary column (30 m × 0.2 mm × 0.25 μm). The column temperature was raised from 50 to 340 °C with a heating rate of 10 °C min^−1^.

CCDC-1879263 (1) contains the supplementary crystallographic data for this paper (ESI9†).

## Conclusions

The compound 1 (4[Ag(py)_2_MnO_4_]·[Ag(py)_4_]MnO_4_) with four [Ag(py)_2_]^+^ and one [Ag(py)_4_]^+^ cations and having four coordinated and one non-coordinated permanganate anion has been synthesized. The compound 1 has several unique structural features including κ^1^O-coordinated permanganates to the silver cations of dimerized [Ag(py)_2_]^+^ units, non-coordinating ionic MnO_4_^−^ and [Ag(py)_4_]^+^ tetrahedra as well as non-linear py–Ag–py moieties resulted from non-equivalent hydrogen bonds between the α-CH of pyridine rings in an [Ag(py)_2_] unit and neighbouring O atoms of the unprecedented κ^1^-O-coordinated MnO_4_^−^. The hydrogen bonds act as reactive centres in the thermally initiated, solid-phase redox reactions between the oxidizing MnO_4_^−^ anion and reducing pyridine ligand. As a result a highly exothermic and explosive reaction, autocatalyzed by the intermediate silver manganese oxides (Körbl's catalysts) the pyridine content is partly oxidized into CO_2_ and H_2_O; the solid phase decomposition end products contain mainly metallic Ag and Mn_3_O_4_ as well as some CO_2_. The enigmatic CO_2_ is an intercalated gas in the manganese oxide matrix instead of the expected chemisorbed carbonate form. Compound 1 is a mild and efficient oxidant toward benzylic alcohols (unsubstituted, 2-MeO, 2-NO_2_ and 4-NO_2_). A solvent- and temperature-dependent oxidation takes place in all reactions with an almost quantitative yield of benzaldehydes. Chloroform is found to be the best solvent. The reaction of benzyl alcohol in CCl_4_ and benzene results in benzaldehyde and benzoic acid, whereas in benzene diphenyl formation occurs due to the oxidative coupling of benzoic acid and benzene in the presence of metallic silver catalyst.

## Conflicts of interest

There are no conflicts to declare.

## Supplementary Material

RA-009-C9RA03230D-s001

RA-009-C9RA03230D-s002
