# Supplementary material for: An unknown component of a selective and mild oxidant: structure and oxidative ability of a double salt-type complex having κ1O-coordinated permanganate anions and three- and four-fold coordinated silver cations
Source: RSC Adv. 2019 Sep 9;9(49):28387–98. doi: 10.1039/c9ra03230d (PMC9071043; doi:10.1039/c9ra03230d)
Supplement: RA-009-C9RA03230D-s001 [file RA-009-C9RA03230D-s001.pdf]

Electronic Supplementary Material (ESI) for RSC Advances.
This journal is © The Royal Society of Chemistry 2019

SUPPORTING INFORMATION

Inorganic Chemistry Frontiers

A selective and mild permanganate oxidant: The first double salt-type complex with three- and four-fold coordinated silver and unique κ^{1}O-coordinated and free-standing permanganates

Gréta Bettina Kovács,[†] Nóra V. May,[†] Petra Alexandra Bombicz,[†] Szilvia Klébert,[†] Péter Németh,[†] Alfréd Menyhárd,[‡],Gyula Novodárszki,[†] Vladimir Petrusevski,[§] Fernanda Paiva Franguelli,[†] József Magyari,[††] Kende Béres,[†] Imre Miklós Szilágyi[¶] and László Kótai[†‖]*

Supporting Information No.1.

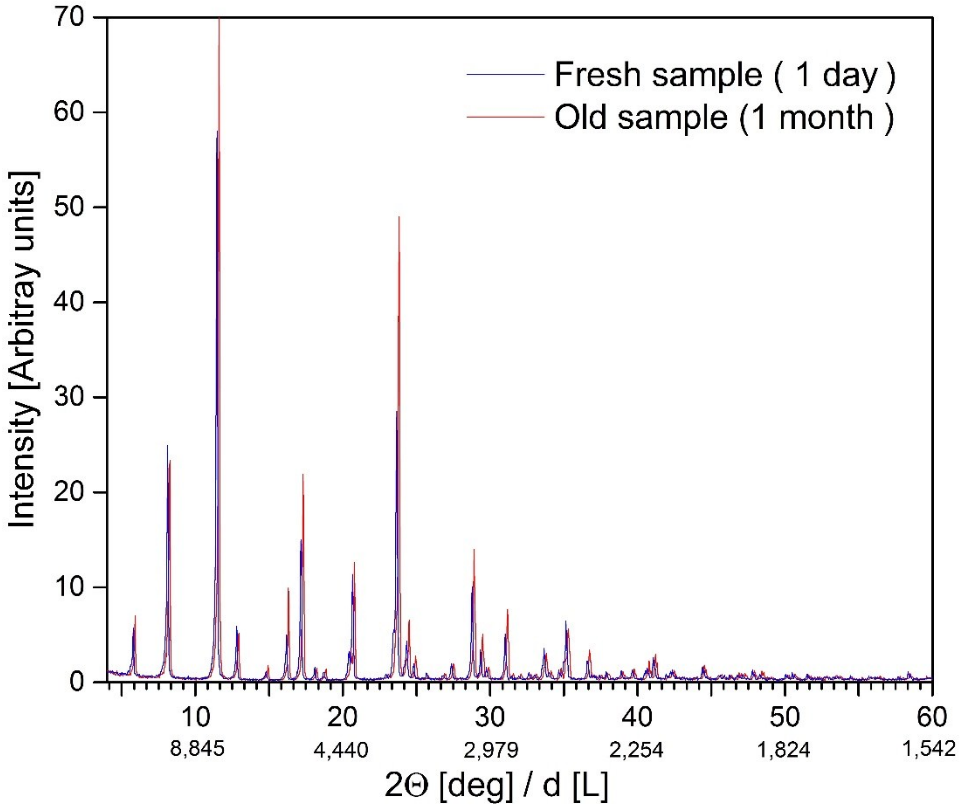

Figure S1/1. XRD of fresh (1 day old) and aged (1 month old) $[4Ag(py)_2MnO_4]\cdot[Ag(py)_4]MnO_4$ (1)

Supporting Information No.2.

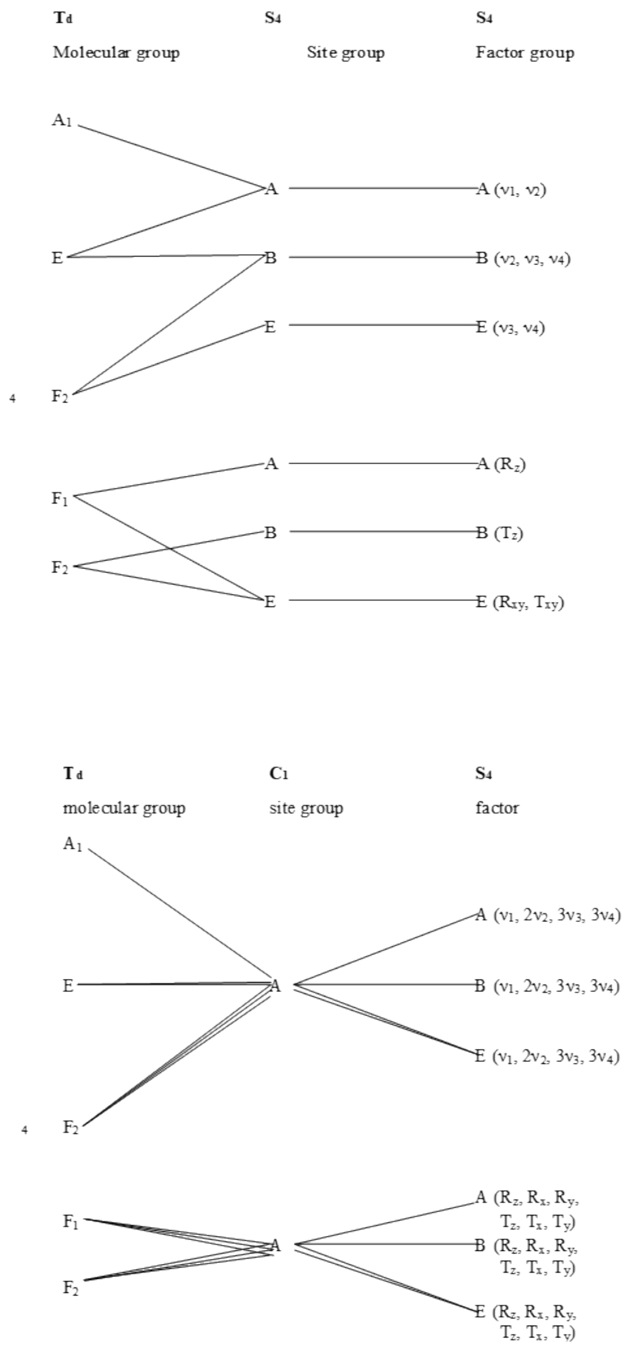

Figure S2/1. Correlation diagram for permanganate anions in the lattice of $4[Agpy_2]MnO_4 \cdot [Agpy_4]MnO_4$. a - S_4 site; b - C_1 site. *R* and *T*- mark hindered rotations and translations of the anions, respectively.

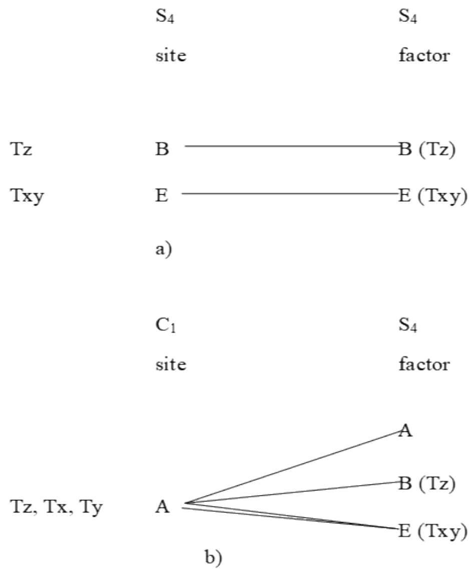

Figure S2/2. Correlation diagrams for the hindered translations of Ag^{+} cations at S_4 sites (a) and those at C_1 sites (b) in compound $[4Ag(py)_2MnO_4]\cdot[Ag(py)_4]MnO_4$.

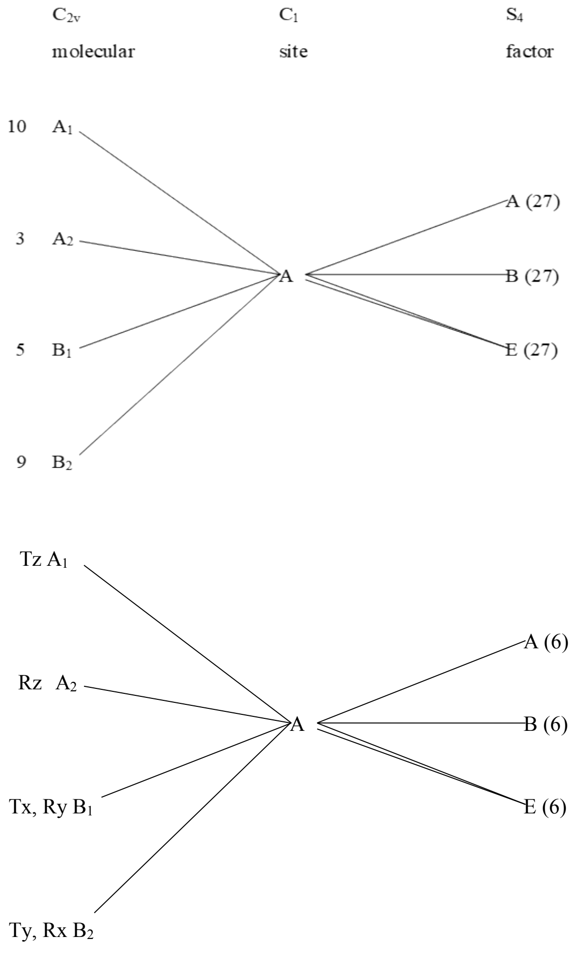

Figure S2/3 . Correlation diagram for pyridine ligand of compound $[4Ag(py)_2MnO_4]\cdot[Ag(py)_4]MnO_4$ (**1**) at C1 site.

Table S2/4. Pyridine ring vibrations and their assignations

C_{2v}	ν	Assignation	IR, Wavenumber, cm^{-1}		Raman shift, cm^{-1} Raman	
			Compound 1	Pyridine	Compound 1	Pyridine
A_1 (in plane)	1	C-H stretch	3102,3091	3077	3081	3076
	2	C-H stretch	3064,3052	3064	3058	3060
	3	C-H stretch	3029,3012	3030	3040,3029	3030
	4	Ring stretch	1596	1596	1577	1578
	5	Ring stretch	1485	1482	1485,1479	1483
	6	C-H wag	1214	1217	1220,1215	1217
	7	C-H wag	1071	1069	1073,1069	1071
	8	Ring bend	1040,1034	1031	1040,1037	1031
	9	Ring breathing	1004	991	861,851	858
	10	Ring bend	-	603	-	601
A_2 (out of plane)	11	C-H wag	986	980	979	982
	12	C-H wag	891,877	884	888	887
	13	Ring bend	-	-	-	-
B_1 (out of plane)	14	C-H wag	1012	996	1010	997
	15	C-H wag	942sh	941	-	-
	16	C-H wag	755	755	-	-
	17	Ring bend	707	707	-	-
	18	Ring bend	-	-	-	-
B_2 (in-plane)	19	C-H stretch	3082,3071	3079	3066	3066
	20	C-H stretch	3039,3029	3039	3030	3030
	21	Ring stretch	1570	1573	1577	1577
	22	Ring stretch	1448,1438	1438	1443	1443
	23	C-H wag	1389	1355	1351	-
	24	Ring stretch	1233,1224	1228	1224	1227
	25	C-H wag	1154	1147	1152	-
	26	C-H wag	-	-	1067,1063	1068
	27	Ring bend	-	653	651	654

Supporting Information No.3.

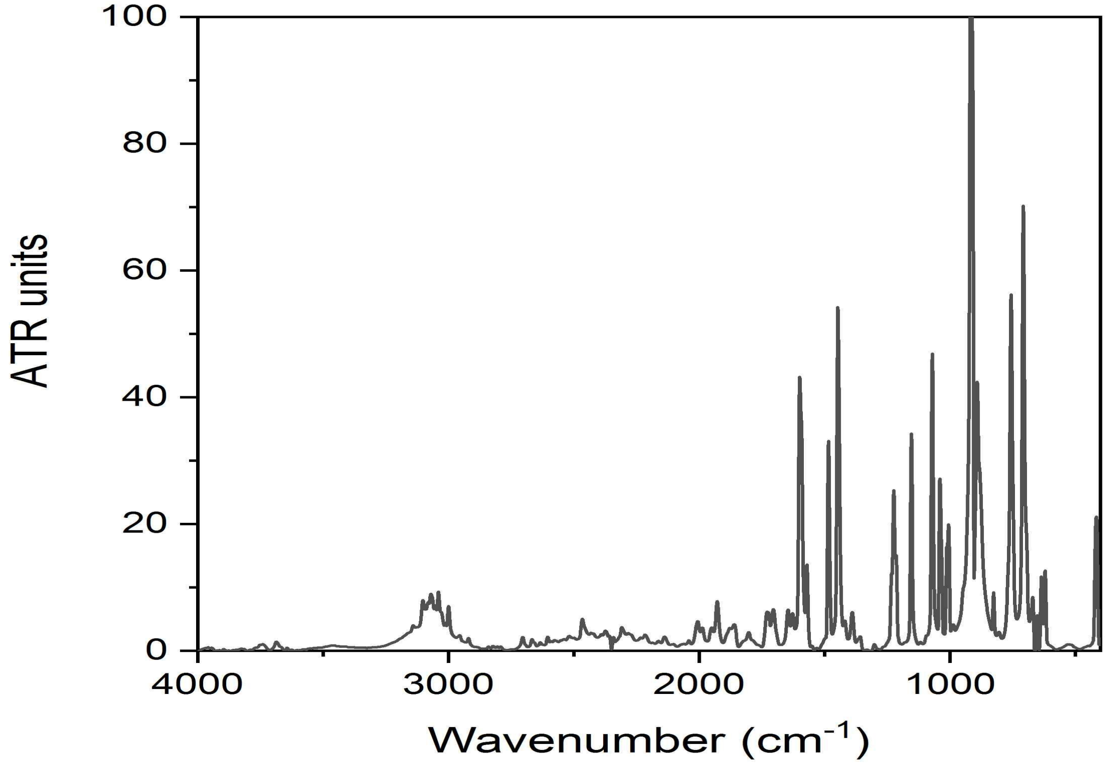

Figure S3/1 . IR spectrum of compound $[4Ag(py)_2MnO_4]\cdot[Ag(py)_4]MnO_4$ (**1**) in ATR mode

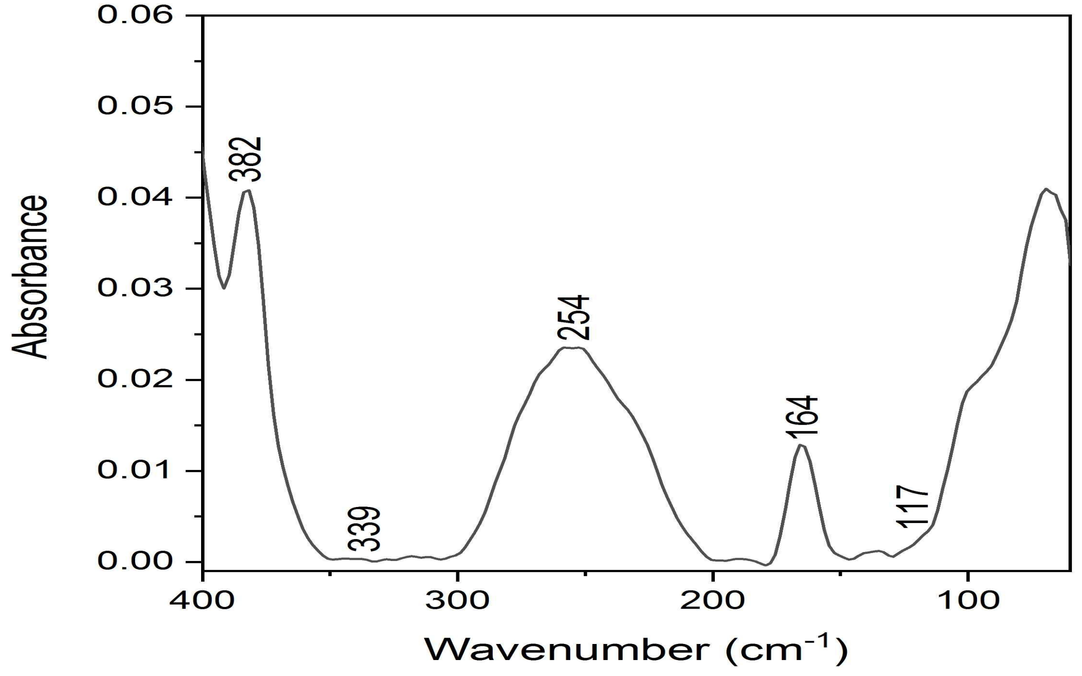

Figure S3/2 .Far-IR spectrum of compound $[4Ag(py)_2MnO_4]\cdot[Ag(py)_4]MnO_4$ (**1**) in polyethylene pellet

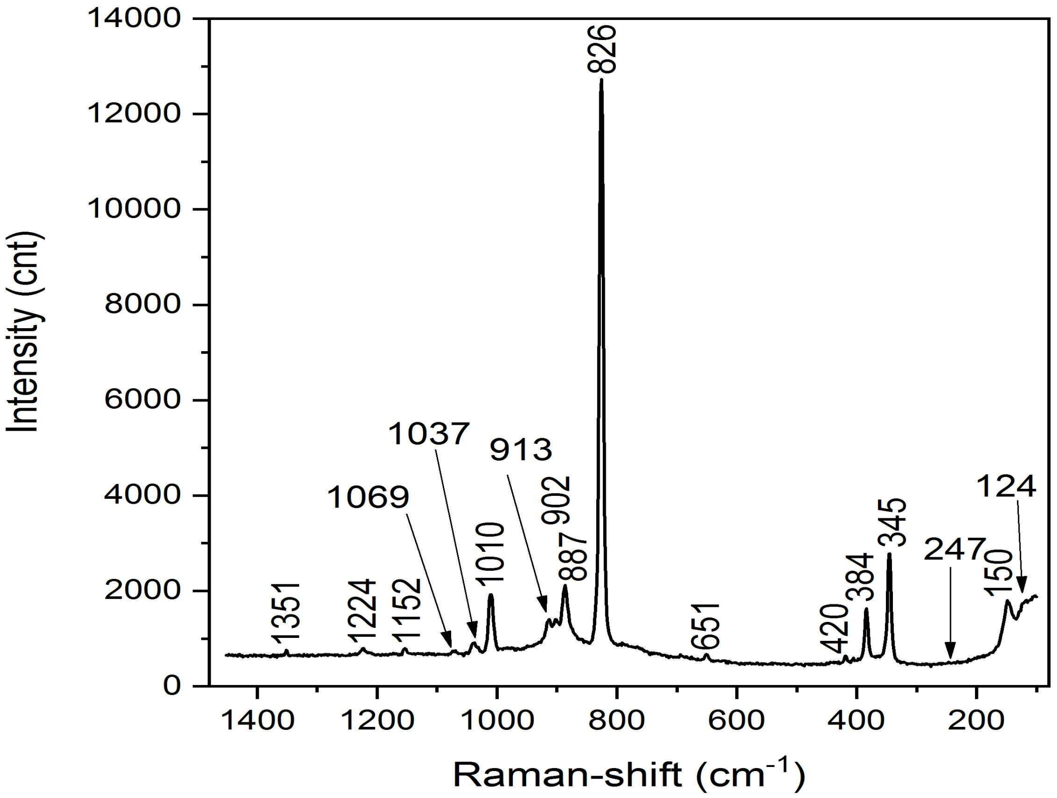

Figure S3/3. Raman spectrum of compound $[4Ag(py)_2MnO_4]{\cdot}[Ag(py)_4]MnO_4$ (**1**) at room temperature

Supporting information . No.4.

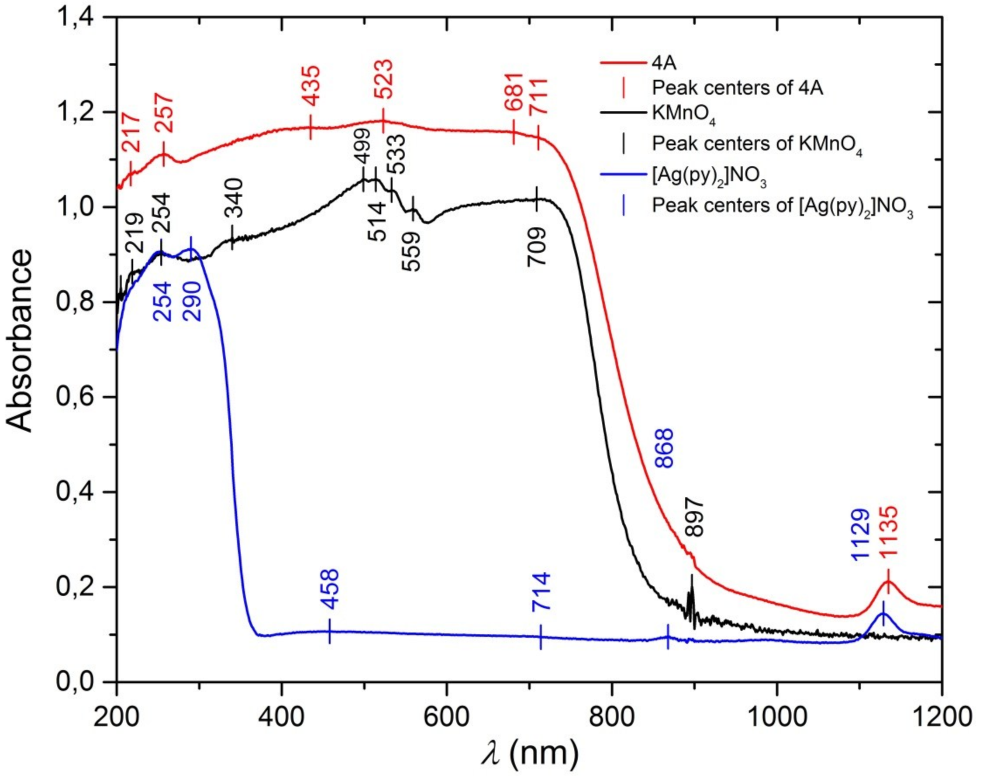

Figure S4/1. UV-Vis spectra of compound $[4Ag(py)_2MnO_4]\cdot[Ag(py)_4]MnO_4$, $KMnO_4$ and $[Ag(py)_2]NO_3$ between 200-1200 nm.

Supporting information No.5.

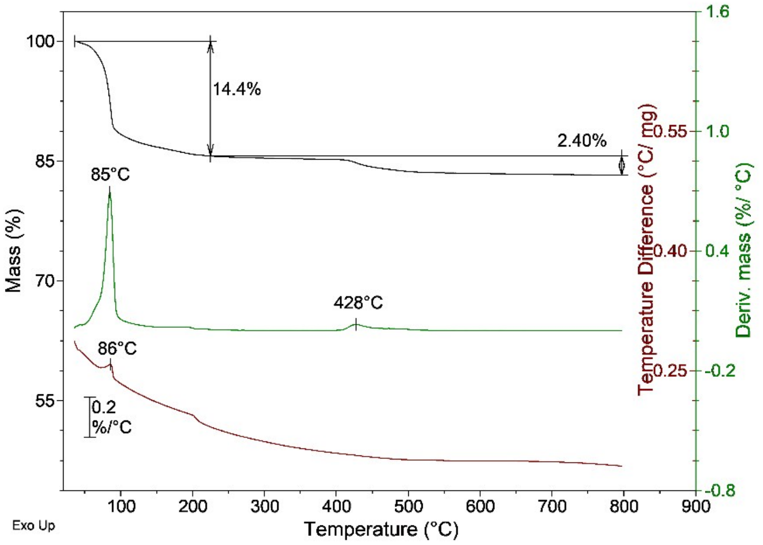

Figure S5/1. Results of TG studies of compound **1** in He atmosphere.

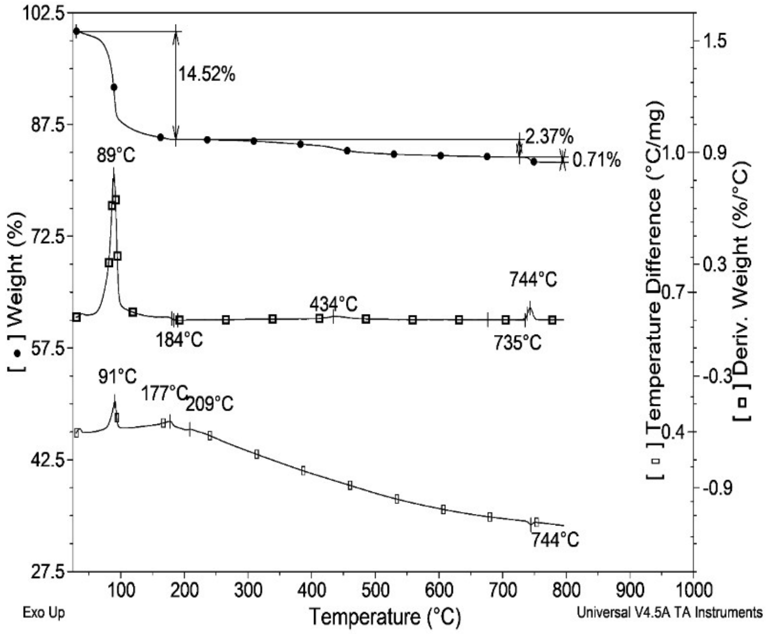

Figure S5/2. TG-DTG-DSC curve of compound $[4Ag(py)_2MnO_4]\cdot[Ag(py)_4]MnO_4$ in air between 25 and 800 °C

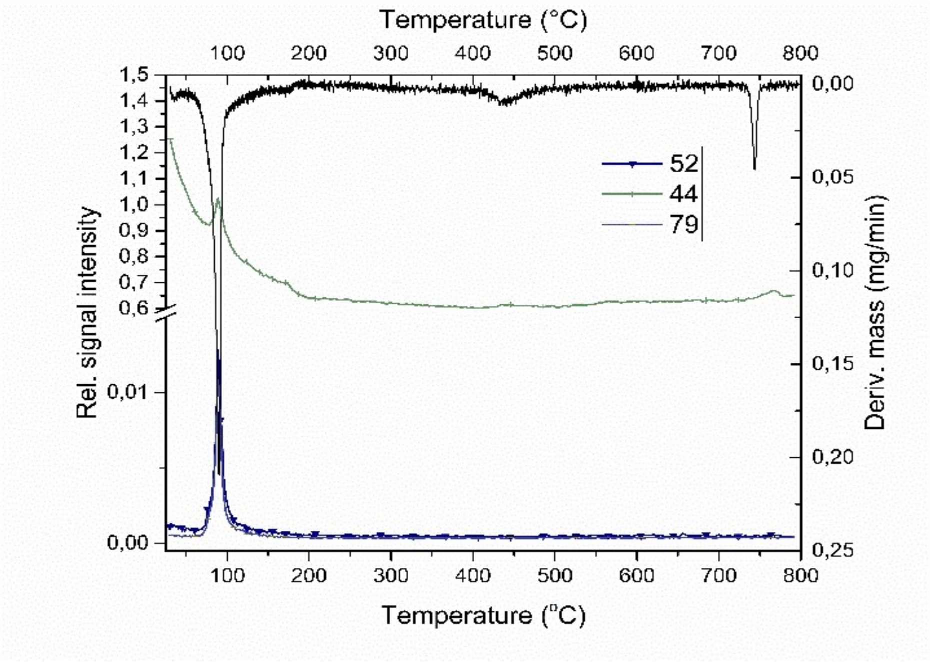

Figure S5/3. Some selected m/z fragment intensities of compound $[4Ag(py)_2MnO_4].[Ag(py)_4]MnO_4$ in air between 25 and 800 °C.

Supporting information No.6.

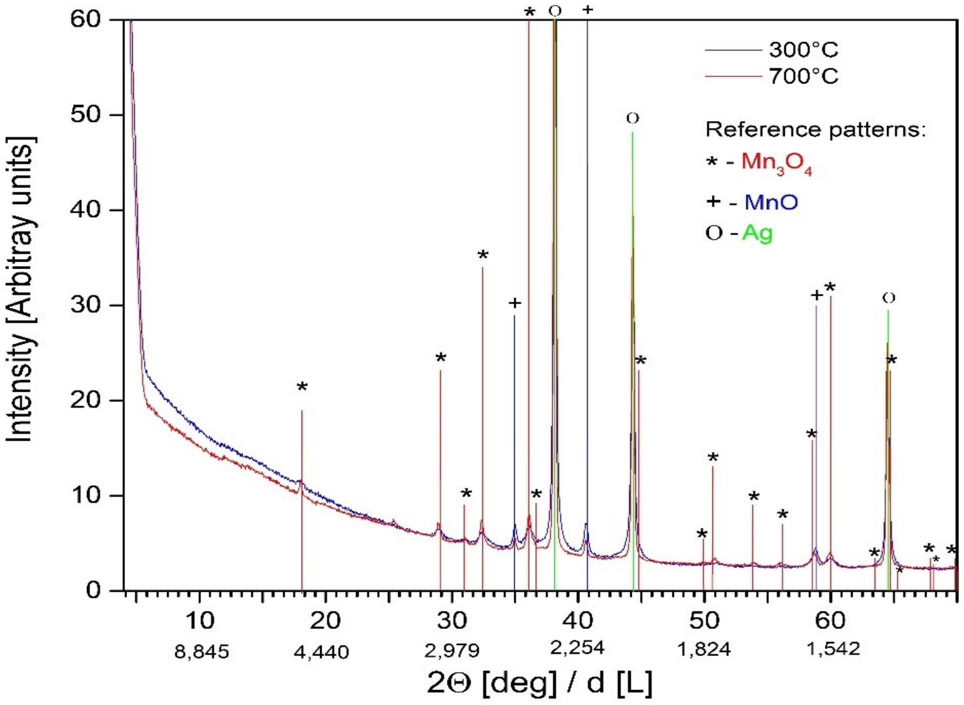

Figure S6/1. XRD of decomposition intermediate (300 °C) and end-product (700 °C) formed from $[4Ag(py)_2MnO_4]\cdot[Ag(py)_4]MnO_4$ under inert atmosphere

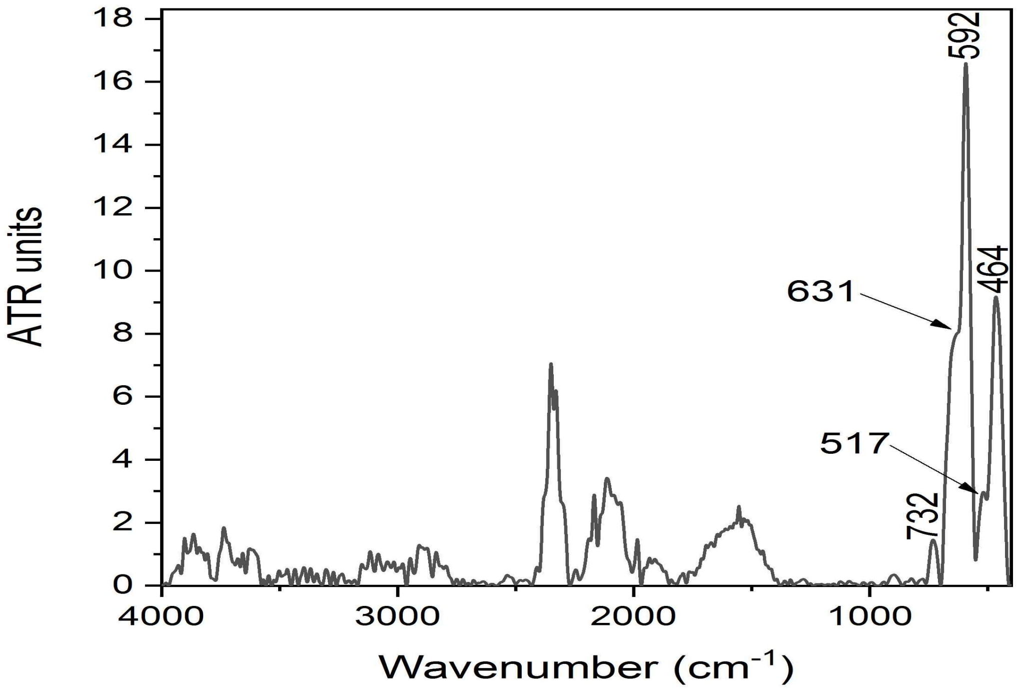

Figure S6/2 . IR spectrum of **I-300** decomposition intermediate formed from compound $[4Ag(py)_2MnO_4]\cdot[Ag(py)_4]MnO_4$ (**1**) at 300 °C in ATR mode

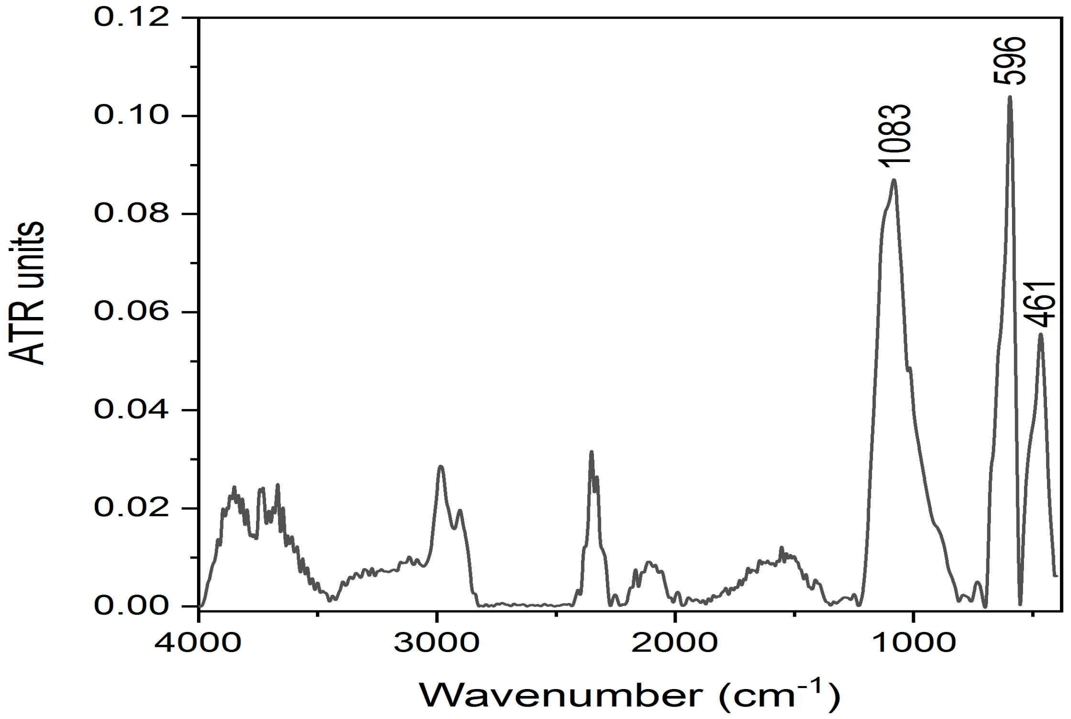

Figure S6/3 IR spectrum of decomposition product formed from compound $[4Ag(py)_2MnO_4]{\cdot}[Ag(py)_4]MnO_4$ (**1**) at 700 °C in ATR mode

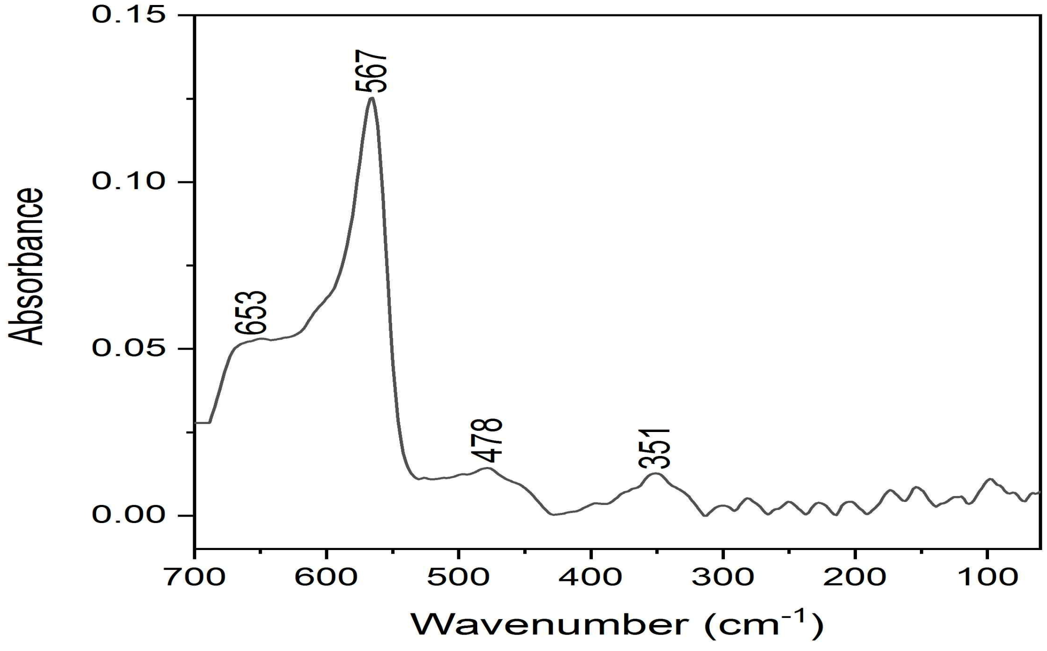

Figure S6/4 . Far-IR spectrum of **I-300** decomposition intermediate formed from compound $[4Ag(py)_2MnO_4]{\cdot}[Ag(py)_4]MnO_4$ (**1**) at 300 °C in polyethylene pellet

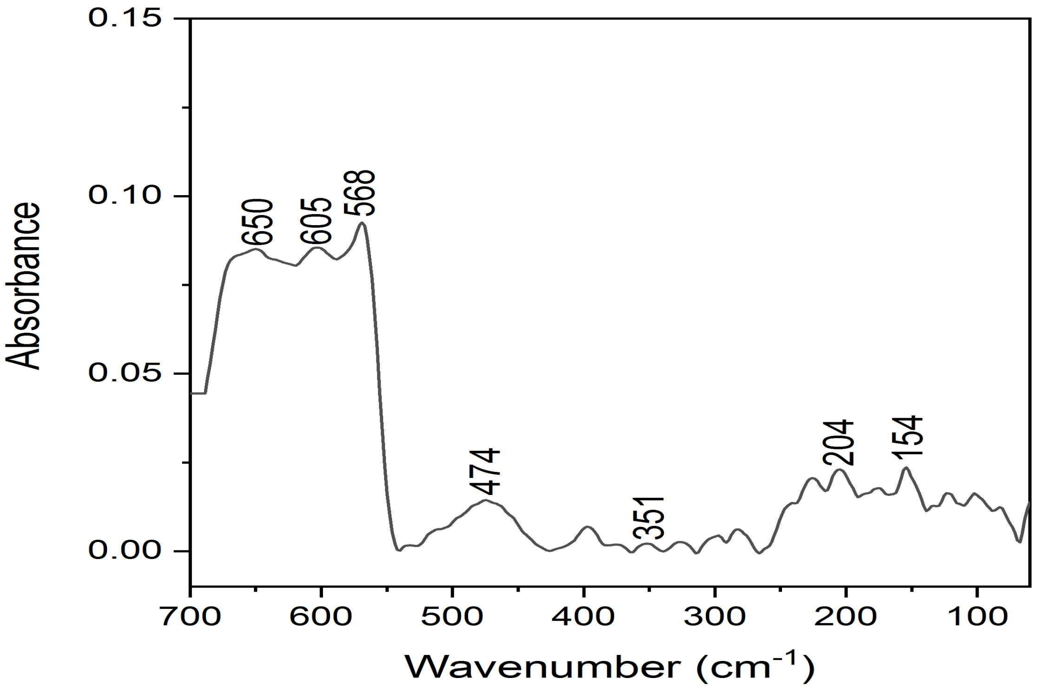

Figure S6/5. Far-IR spectrum of decomposition product formed from compound $[4Ag(py)_2MnO_4]\cdot[Ag(py)_4]MnO_4$ (**1**) at 700 °C in polyethylene pellet

Supporting information No.7

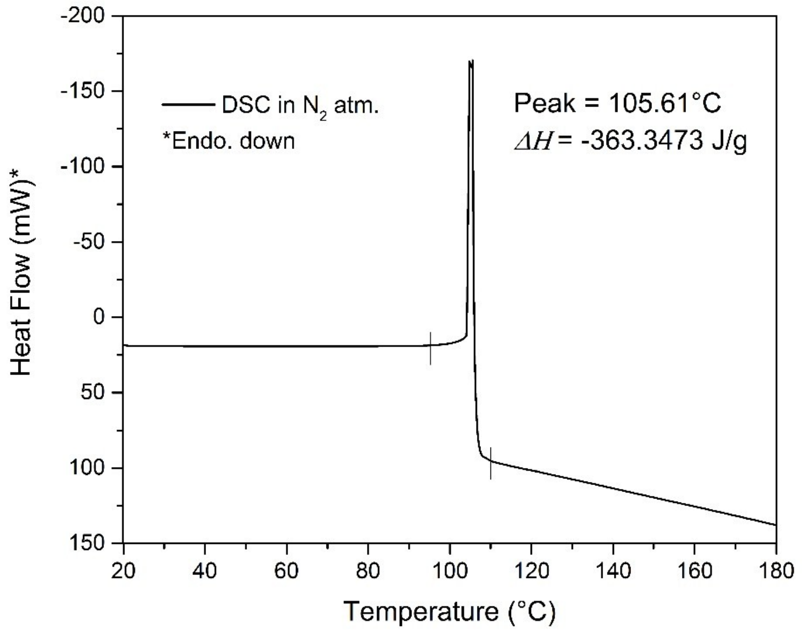

Figure S7/1. DSC of fresh $[4Ag(py)_2MnO_4]\cdot[Ag(py)_4]MnO_4$ under N_2

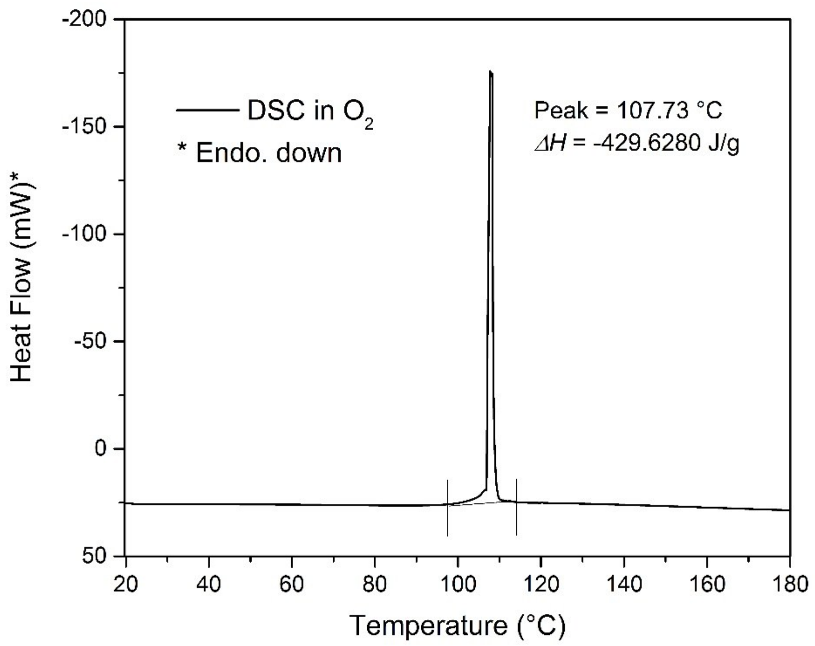

Figure S7/2. DSC of fresh $[4Ag(py)_2MnO_4]\cdot[Ag(py)_4]MnO_4$ under O_2

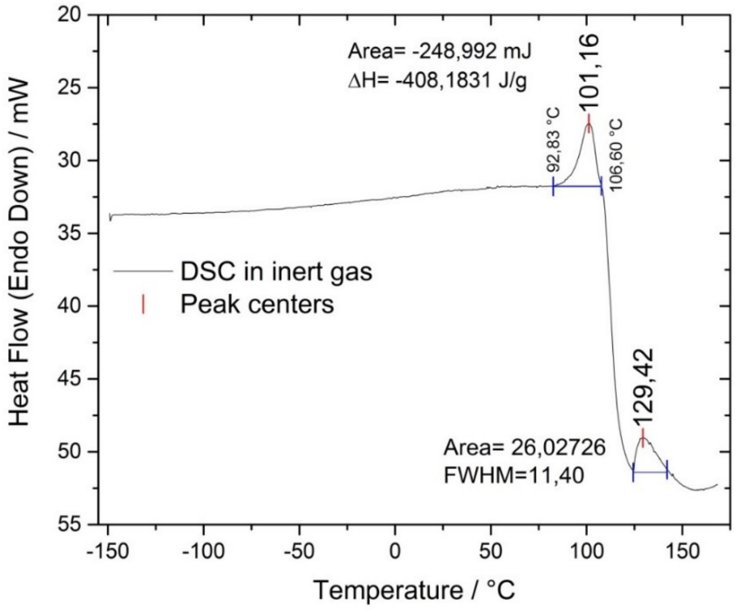

Figure S7/3. DSC of the aged (1 month old) compound $[4Ag(py)_2MnO_4]\cdot[Ag(py)_4]MnO_4$ under N_2 between -150 and 170 °C

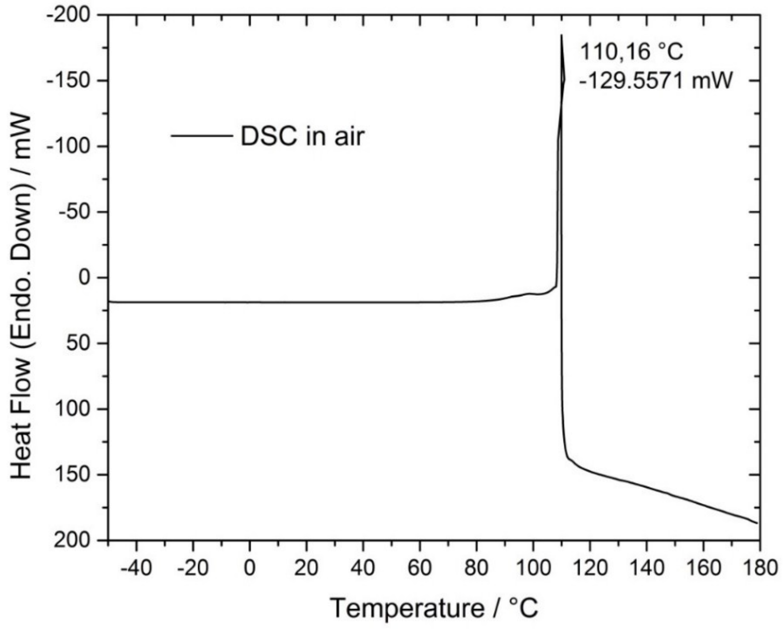

Figure S7/4. DSC of the aged (1 month old) compound $[4Ag(py)_2MnO_4]\cdot[Ag(py)_4]MnO_4$ under air between -50 and 170 °C

Supporting Information No.8.

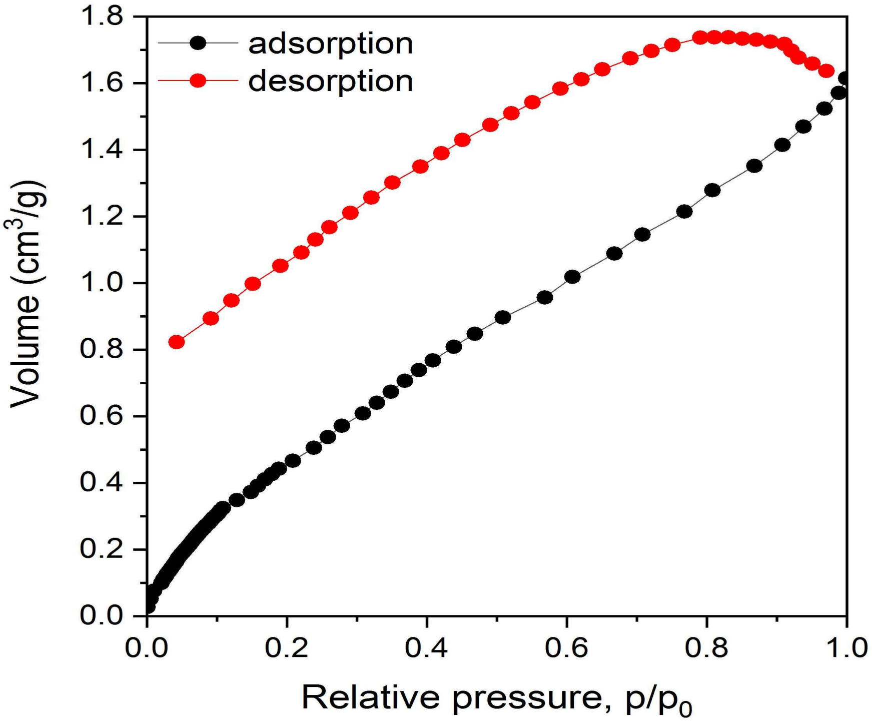

Figure S8/1. CO_2 soprtion-desorption isotherms of I-300 (decomposition intermediate of $[4Ag(py)_2MnO_4]\cdot[Ag(py)_4]MnO_4$ at 300 °C under N_2

Supporting information No. 9.

The $4[Ag(py)_2]MnO_4\cdot[Ag(py)_4]MnO_4$ (**1**) was crystallised from pyridine solution of $AgMnO_4$ by adding 10-fold amount of water and left to crystallize the solution at room temperature.
1 crystallises in the tetragonal crystal system, space group *I*-4.
The diffraction pattern of the black, block type single crystal of **1** with the size of 0.25 x 0.25 x 0.20 mm was measured on a Rigaku RAxis Rapid II diffractometer at room temperature using MoK_{α} radiation.
There is one $[Ag(py)_2MnO_4]$ and a quarter of $[Ag(py)_4]MnO_4$ in the asymmetric unit of **1** (Figure S9/1). The Kitaigorodskii packing coefficient is 69.6 %. There is no residual solvent accessible void in the crystal lattice.
The atomic positions were determined by direct methods, hydrogen atoms were placed into calculated positions. Crystal data and details of the structure determination and refinement are listed in Table S9/10, atomic coordinates and equivalent isotropic displacement parameters are in Table S9/11, hydrogen coordinates and equivalent isotropic displacement parameters are in Table S9/12, anisotropic displacement parameters are listed in Table S9/13, while bond length and angles can be found in Table S9/14 and S9/15, respectively.

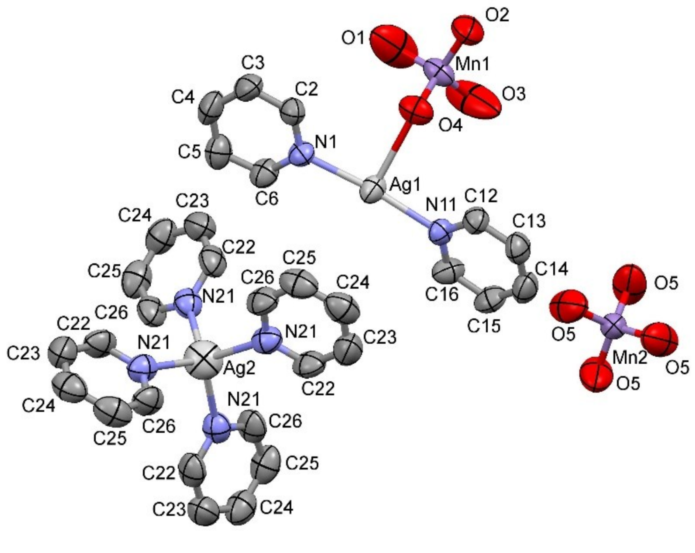

Figure S9/1 ORTEP presentation of the molecular structure and atomic numbering scheme of compound **1**. The asymmetric unit contains $[Ag(py)_2]MnO_4$ and $[Ag(py)_4]MnO_4$ in the stoichiometric ratio of 1 : ¼. The displacement ellipsoids are drawn at the 50% probability level.

The conformation of the $[Agpy_2MnO_4]$ moiety is shown in Figure S9/2. The angle of the two pyridine rings is 12.03°. The salt is forming a chain along the 'c' crystallographic axis (Figure S9/3). These chains are arranged in a framework structure presented in Figure S9/4a.

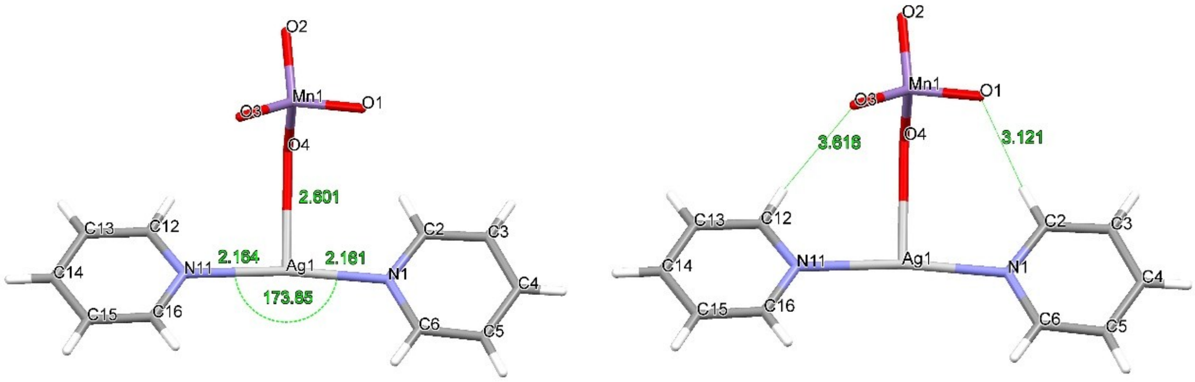

Figure S9/2 a., The $[Agpy_2MnO_4]$ moiety in **1** showing the bond distances and angle around the Ag^+ (see also Table S8/14 and S9/15). b., The Cα-H…$O_{permanganate}$ interactions are weak, they slightly contribute to the complex stability.

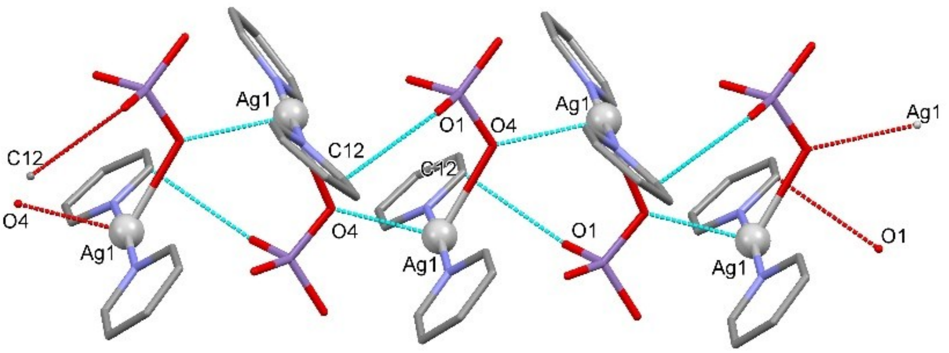

Figure S9/3. The chain formed by $[Agpy_2MnO_4]$ along the 'c' crystallographic axis in **1**.

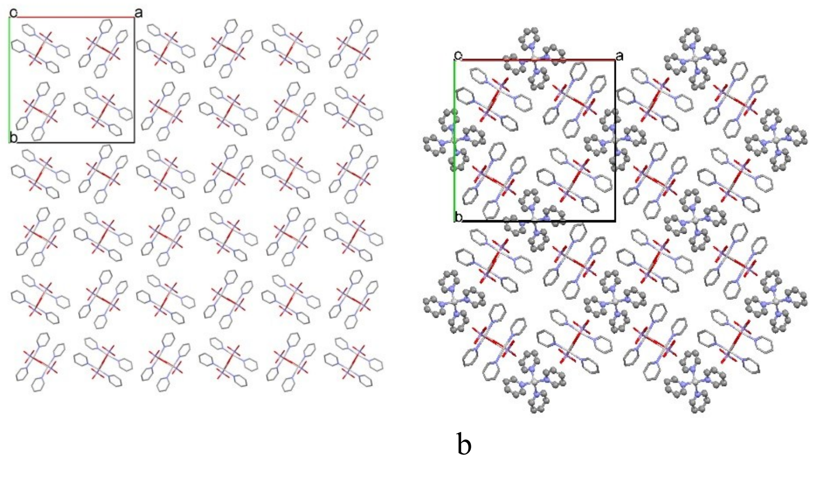

a b

S9/4 a., The framework constructed by the chains of $[Agpy_2]MnO_4$ viewing along the 'c' crystallographic axis in **1**. b., The framework constructed by $[Agpy_2MnO_4]$ and $[Agpy_4]^+$ having Mn1-O2… π interaction between them in **1**. The voids of the figure are filled with the

MnO_4^- anion.

The conformation of the $[Agpy_4]^+$ is presented in Figure S9/5. Quarter of the molecule is in the asymmetric unit. The Ag^+ cation is placed on a 4-fold rotoinversion axis. The $[Agpy_2MnO_4]$ chains and the $[Agpy_2MnO_4]$ complexes are connected by a Mn1-O2… π interactions (Figure S9/6), where Mn1-O2…Cg(py) [1/2-y, -1/2+x, ½-z] distance is 3.469(7) Å, their angle is 161.0(3)°. The framework constructed by the two interacting organic ligand complexes is shown on Figure S9/4b. There is neither classical hydrogen bond, nor π...π interaction in the crystal structure. The packing arrangement in crystal **1** viewing from the a, b and c crystallographic axes is shown in Figures S9/7.

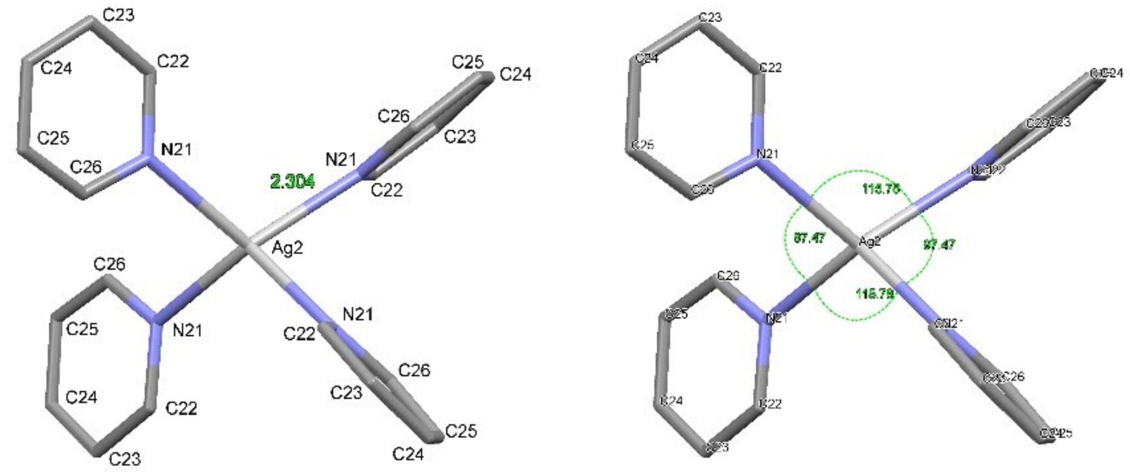

Figure S9/5 The conformation of the $[Agpy_4]^+$ a., the Ag..N distance and b., the angles around the central cation in **1**.

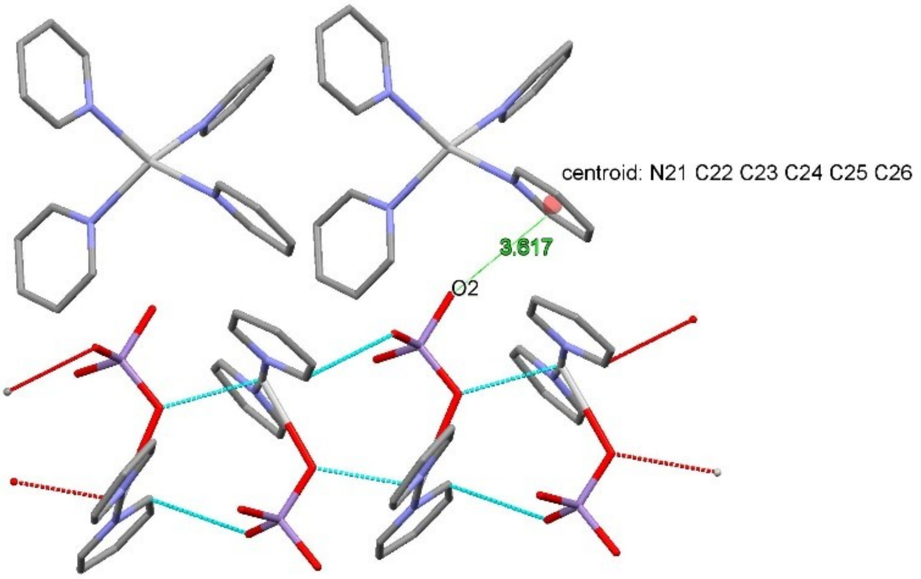

Figure S9/6 The $[Agpy_2MnO_4]$ chains and the $[Agpy_2]MnO_4$ complexes connected by Mn1-O2…π interactions in **1**.

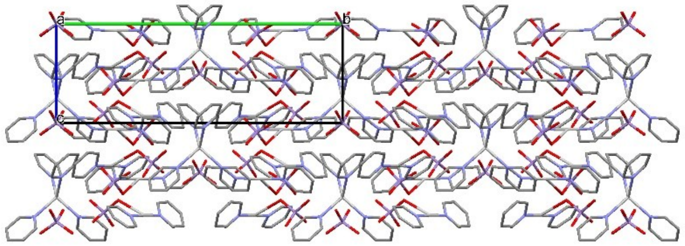

a.,

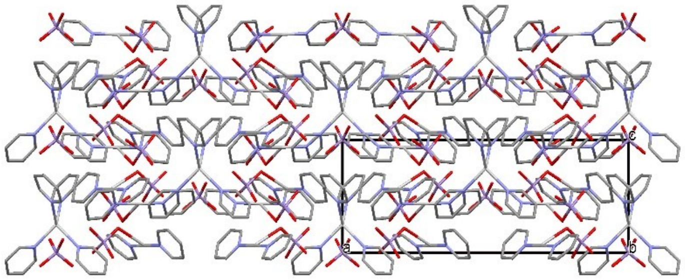

b.,

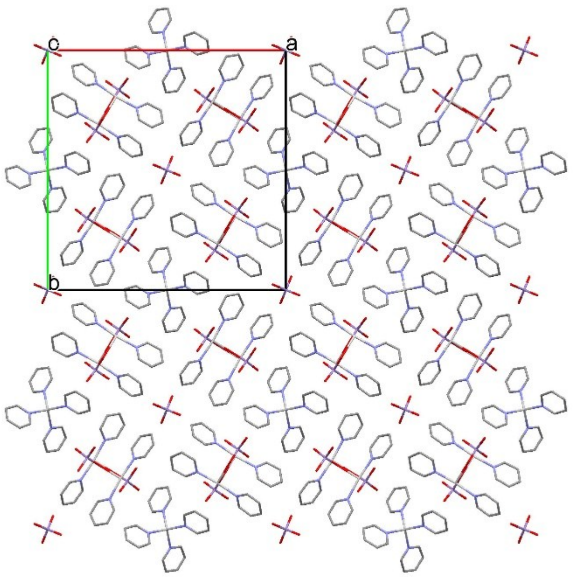

c.,

Figure S9/7 The packing arrangement in the crystal of **1.** a., View from the a crystallographic axis. b., View from the b crystallographic axis. Views a and b are identical owned to the space group symmetry. c., View from the c crystallographic axis.

The structure of the perchlorate analogue of **1**, $4[Ag(py)_2]ClO_4.[Ag(py)_4]ClO_4$, (DITCEO, **1-ClO$_4$**), has already been reported [DITCEO]. DITCEO crystallises in the tetragonal crystal system, space group I-4, the cell parameters a=21.95(1) b=21.95(1) c=7.684(3), $\alpha=\beta=\gamma=90^o$. The two crystals are isostructural, the cell similarity index π=0.00044, the unit cell of DITCEO is larger with 31 Å^3, 0.85% than the unit cell of **1**. Placement of the $[Agpy_4]^+$ cations slightly differ in the two crystal lattices, it is compared in Figure S9/8.

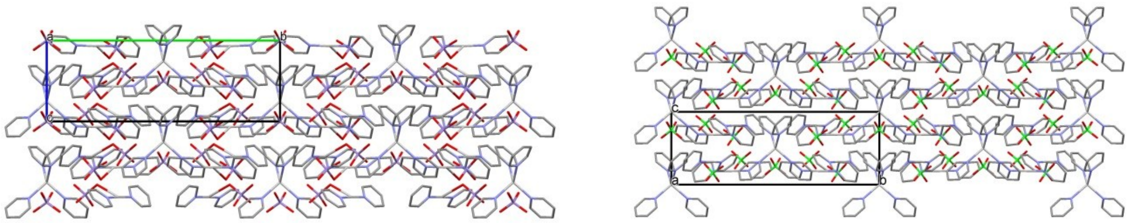

a., b.,

Figure S9/8 Crystal packing arrangements of a., **1** and b., DITCEO (**1-ClO$_4$**) viewing from the 'a' crystallographic directions.

The crystal structure of [Ag(py)$_2$MnO$_4$].0.5py (**4**) was recently reported by us. Comparison of the conformational arrangement of the [Ag(py)$_2$MnO$_4$] salt in **1** and **4** is presented in Fig. S9/9. The different molecular geometry is characterized by the rmsd value of 0.7805 and the max$_D$ value of 1.3312Å.

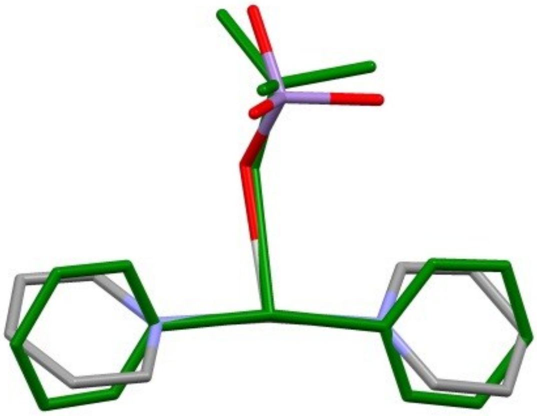

Figure S9/9 Comparison of the [Ag(py)$_2$MnO$_4$] moieties from the crystal structures of **1** (coloured by elements) and [Ag(py)$_2$MnO$_4$]·0.5py (**4)** (green).

Experimental:

Crystal data of **1**: $C_{60}H_{60}Ag_5Mn_5N_{12}O_{20}$, *Fwt.*: 2083.25, black, block, size: 0.25 x 0.25 x 0.2 mm, tetragonal, space group *I* -4, a = 21.982(3)Å, b = 21.982(3)Å, c = 7.5974(15)Å, α = 90°, β = 90°, γ = 90°, V = 3671.1(13)Å^3, T = 293(2)K, Z= 2, F(000) = 2048, D_x = 1.885 Mg/m^3, μ 2.207mm^{-1}.

A crystal of **1** was mounted on a loop. Cell parameters were determined by least-squares using 30410 (3.39 ≤ θ ≤ 25.28°) reflections. Intensity data were collected on a Rigaku RAxis Rapid II diffractometer (monochromator; Mo-K_α radiation, λ = 0.71073Å) at 293(2) K in the range 3.384 ≤ θ ≤ 25.242. A total of 35359 reflections were collected of which 3325 were unique [R(int) = 0.0489, $R(\sigma)$ = 0.0244]; intensities of 3004 reflections were greater than 2σ(I). Completeness to θ = 0.997.The crystal contains two Ag complex cations with pyridine molecules as ligands and two permanganate anions. The ratio of the two complexes is 1:4 in the double salt. The lattice has the high I-4 symmetry. It results in low data to parameter ratio. In case of one tetrahedral cation and one tetrahedral anion there is only one-fourth of the molecule in the asymmetric unit. Numerical absorption correction was applied to the data (the minimum and maximum transmission factors were 0.8567 and 0.9965).

The structure was solved by direct methods. Anisotropic full-matrix least-squares refinement on F^2 for all non-hydrogen atoms yielded $R1$ = 0.0367 and $wR2$ = 0.0655 for 1332 [I>2σ(I)] and $R1$ = 0.0441 and $wR2$ = 0.0675 for all (3325) intensity data, (number of parameters = 230, goodness-of-fit = 1.080, the maximum and mean shift/esd is 0.000 and 0.000). The absolute structure parameter is 0.010(10). (Friedel coverage: 0.821, Friedel fraction max.: 0.998, Friedel fraction full: 0.998). The maximum and minimum residual electron density in the final difference map was 0.526 and -0.324e e.Å^{-3}. The weighting scheme applied was w = $1/[\sigma^2(F_o^2)+(0.02940.0000P)^2+0.0000P]$ where $P = (F_o^2+2F_c^2)/3$.

Hydrogen atomic positions were calculated from assumed geometries. Hydrogen atoms were included in structure factor calculations but they were not refined. The isotropic displacement parameters of the hydrogen atoms were approximated from the U(eq) value of the atom they were bonded to.

Table S9/10. Crystal data and details of the structure determination and refinement of **1**.

Empirical formula	C_{60} H_{60} Ag_5 Mn_5 N_{12} O_{20}
Formula weight	2083.25
Temperature	293(2) K
Radiation and wavelength	Mo-Kα, λ =0.71073Å
Crystal system	tetragonal
Space group	I -4
Unit cell dimensions	a =21.982(3)Å
	b =21.982(3)Å
	c =7.5974(15)Å
	α =90°, β =90°, γ =90°
Volume	3671.1(13)$Å^3$
Z	2
Density (calculated)	1.885 Mg/m^3
Absorption coefficient, μ	2.207 mm^{-1}
F(000)	2048
Crystal colour	black
Crystal description	block
Crystal size	0.25 x 0.25 x 0.2 mm
Absorption correction	numerical
Max. and min. transmission	0.85670.9965
θ–range for data collection	3.389 ≤ θ ≤ 25.244°
Index ranges	-25 ≤ h ≤26;-26≤ k ≤26;-9 ≤ l ≤9
Reflections collected	35359
Completeness to 2θ	0.997
Absolute structure parameter	0.015(9)
Friedel coverage	0.821
Friedel fraction max.	0.998
Friedel fraction full	0.998
Independent reflections	3325 [R(int) =0.0489
Reflections I>2□(I)	3004
Refinement method	full-matrix least-squares on F^2
Data / restraints / parameters	3325 /0 /230
Goodness-of-fit on F^2	1.080
Final R indices [I>2σ(I)]	$R1$ =0.0367, $wR2$ =0.0655
R indices (all data)	$R1$ =0.0441, $wR2$ =0.0675
Max. and mean shift/esd	0.000;0.000
Largest diff. peak and hole	0.526;-0.324 e.$Å^{-3}$

Table S9/11. Atomic coordinates (x 10^4) and equivalent isotropic displacement parameters ($Å^2$ x 10^3) of **1**. *U*(eq) is defined as one third of the trace of the orthogonalized U_{ij} tensor.

Ag2	10000	0	5000	101(1)
Mn2	10000	5000	-2500	54(1)
Mn1	8345.0(5)	2011.2(5)	6537(2)	59(1)
O1	8713(4)	2548(4)	7470(13)	165(5)
Ag1	8065.7(2)	2130.8(2)	1835.0(8)	60(1)
N1	8617(2)	2945(2)	1838(8)	50(1)
N11	7599(2)	1266(2)	1921(9)	53(1)
O4	7841(2)	2319(3)	5317(7)	72(2)
C14	6990(4)	171(4)	2064(15)	79(3)
C2	9066(3)	3032(4)	2982(12)	64(2)
C6	8533(3)	3375(3)	607(11)	61(2)
C12	7789(4)	807(3)	2954(12)	64(2)
C16	7102(3)	1167(3)	959(11)	60(2)
C3	9438(4)	3538(4)	2941(13)	71(2)
C13	7502(4)	253(3)	3045(13)	72(2)
C15	6791(4)	629(4)	985(13)	74(2)
C4	9350(4)	3962(3)	1643(15)	72(2)
O5	10223(3)	5550(3)	-3649(12)	119(3)
C5	8897(4)	3882(4)	482(12)	69(2)
N21	9228(3)	-152(3)	7004(10)	64(2)
C22	9272(4)	-598(4)	8175(15)	78(2)
C26	8716(4)	169(4)	7044(14)	76(2)
C24	8308(5)	-433(5)	9331(14)	85(3)
C23	8831(5)	-757(4)	9328(13)	83(3)
C25	8244(4)	39(4)	8203(18)	91(3)
O3	8812(3)	1650(3)	5361(9)	88(2)
O2	8039(4)	1586(5)	7893(15)	187(5)
Ag2	10000	0	5000	101(1)

Table S9/12 Hydrogen coordinates (x 10^4) and isotropic displacement parameters (Å^2 x 10^3) of **1**.

	x	*y*	*z*	*U*(iso)
H14	6777	-194	2125	95
H2	9132	2740	3847	76
H6	8217	3328	-192	73
H12	8133	868	3643	77
H16	6960	1478	237	72
H3	9742	3590	3778	85
H13	7653	-56	3755	86
H15	6450	575	281	89
H4	9601	4301	1568	86
H5	8830	4167	-401	83
H22	9633	-817	8205	94
H26	8671	493	6267	91
H24	7994	-535	10100	102
H23	8887	-1080	10101	100
H25	7892	272	8203	110

Table S9/13 Anisotropic displacement parameters (Å^2 x 10^3). The anisotropic displacement factor exponent takes the form: $-2\pi^2(h^2a^{*2}U_{11} + ... + 2hka^*b^*U_{12})$ of **1**.

	U_{11}	U_{22}	U_{33}	U_{23}	U_{13}	U_{12}
Ag2	119(1)	119(1)	65(1)	0	0	0
Mn2	51(1)	51(1)	59(2)	0	0	0
Mn1	63(1)	72(1)	43(1)	1(1)	1(1)	21(1)
O1	132(6)	189(8)	173(10)	-119(8)	-75(6)	47(6)
Ag1	64(1)	55(1)	62(1)	3(1)	-5(1)	-15(1)
N1	52(3)	53(3)	46(3)	7(3)	-1(3)	-5(2)
N11	55(3)	52(3)	51(4)	-5(3)	-5(3)	-5(2)
O4	69(3)	91(4)	56(4)	9(3)	-2(3)	15(3)
C14	83(6)	60(5)	94(7)	0(5)	12(6)	-22(4)
C2	58(4)	79(5)	53(5)	17(4)	-4(4)	-9(4)
C6	66(4)	53(5)	63(5)	0(4)	-11(4)	-1(4)
C12	72(5)	66(5)	55(5)	1(4)	-10(4)	-6(4)
C16	56(4)	57(5)	68(5)	-11(4)	-8(4)	5(3)
C3	63(5)	84(6)	66(6)	-11(5)	-13(4)	-17(4)
C13	91(6)	56(4)	68(6)	9(4)	7(5)	-9(4)
C15	55(4)	69(6)	97(7)	-22(5)	-3(4)	-13(4)
C4	72(5)	56(4)	88(7)	1(5)	14(5)	-12(4)
O5	83(4)	119(5)	156(8)	76(6)	16(4)	1(4)
C5	78(5)	51(5)	77(6)	13(4)	-3(5)	-2(4)
N21	71(4)	62(4)	58(4)	3(4)	-8(3)	3(3)
C22	83(6)	65(5)	88(6)	14(5)	-21(6)	11(4)
C26	86(6)	61(5)	80(6)	12(5)	-17(6)	6(4)
C24	92(7)	83(6)	81(7)	-14(6)	13(5)	-20(6)
C23	109(8)	77(6)	65(6)	11(5)	-15(6)	-15(6)
C25	73(6)	81(6)	120(9)	-12(7)	-12(7)	10(5)
O3	77(4)	78(4)	109(5)	-12(4)	28(4)	13(3)
O2	147(7)	227(10)	188(10)	145(9)	103(7)	93(7)

Table S9/12 Hydrogen coordinates (x 10^4) and isotropic displacement parameters (Å^2 x 10^3) of **1**.

	x	*y*	*z*	*U*(iso)
H14	6777	-194	2125	95
H2	9132	2740	3847	76
H6	8217	3328	-192	73
H12	8133	868	3643	77
H16	6960	1478	237	72
H3	9742	3590	3778	85
H13	7653	-56	3755	86
H15	6450	575	281	89
H4	9601	4301	1568	86
H5	8830	4167	-401	83
H22	9633	-817	8205	94
H26	8671	493	6267	91
H24	7994	-535	10100	102
H23	8887	-1080	10101	100
H25	7892	272	8203	110

Table S9/13 Anisotropic displacement parameters (Å^2 x 10^3). The anisotropic displacement factor exponent takes the form: $-2\pi^2(h^2a^{*2}U_{11} + ... + 2hka^*b^*U_{12})$ of **1**.

	U_{11}	U_{22}	U_{33}	U_{23}	U_{13}	U_{12}
Ag2	119(1)	119(1)	65(1)	0	0	0
Mn2	51(1)	51(1)	59(2)	0	0	0
Mn1	63(1)	72(1)	43(1)	1(1)	1(1)	21(1)
O1	132(6)	189(8)	173(10)	-119(8)	-75(6)	47(6)
Ag1	64(1)	55(1)	62(1)	3(1)	-5(1)	-15(1)
N1	52(3)	53(3)	46(3)	7(3)	-1(3)	-5(2)
N11	55(3)	52(3)	51(4)	-5(3)	-5(3)	-5(2)
O4	69(3)	91(4)	56(4)	9(3)	-2(3)	15(3)
C14	83(6)	60(5)	94(7)	0(5)	12(6)	-22(4)
C2	58(4)	79(5)	53(5)	17(4)	-4(4)	-9(4)
C6	66(4)	53(5)	63(5)	0(4)	-11(4)	-1(4)
C12	72(5)	66(5)	55(5)	1(4)	-10(4)	-6(4)
C16	56(4)	57(5)	68(5)	-11(4)	-8(4)	5(3)
C3	63(5)	84(6)	66(6)	-11(5)	-13(4)	-17(4)
C13	91(6)	56(4)	68(6)	9(4)	7(5)	-9(4)
C15	55(4)	69(6)	97(7)	-22(5)	-3(4)	-13(4)
C4	72(5)	56(4)	88(7)	1(5)	14(5)	-12(4)
O5	83(4)	119(5)	156(8)	76(6)	16(4)	1(4)
C5	78(5)	51(5)	77(6)	13(4)	-3(5)	-2(4)
N21	71(4)	62(4)	58(4)	3(4)	-8(3)	3(3)
C22	83(6)	65(5)	88(6)	14(5)	-21(6)	11(4)
C26	86(6)	61(5)	80(6)	12(5)	-17(6)	6(4)
C24	92(7)	83(6)	81(7)	-14(6)	13(5)	-20(6)
C23	109(8)	77(6)	65(6)	11(5)	-15(6)	-15(6)
C25	73(6)	81(6)	120(9)	-12(7)	-12(7)	10(5)
O3	77(4)	78(4)	109(5)	-12(4)	28(4)	13(3)
O2	147(7)	227(10)	188(10)	145(9)	103(7)	93(7)

Table S9/14. Bond lengths [Å] in **1**.

Ag2-N21#1	2.305(7)	Ag2-N21#2	2.305(7)
Ag2-N21	2.305(7)	Ag2-N21#3	2.305(7)
Mn2-O5#4	1.570(6)	Mn2-O5#5	1.570(6)
Mn2-O5#6	1.570(6)	Mn2-O5	1.570(6)
Mn1-O2	1.545(8)	Mn1-O3	1.576(6)
Mn1-O1	1.596(8)	Mn1-O4	1.596(5)
Ag1-N11	2.162(5)	Ag1-N1	2.163(5)
N1-C2	1.328(9)	N1-C6	1.342(9)
N11-C16	1.332(9)	N11-C12	1.344(9)
C14-C13	1.36(1)	C14-C15	1.37(1)
C2-C3	1.38(1)	C6-C5	1.37(1)
C12-C13	1.37(1)	C16-C15	1.36(1)
C3-C4	1.37(1)	C4-C5	1.34(1)
N21-C22	1.33(1)	N21-C26	1.33(1)
C22-C23	1.35(1)	C26-C25	1.39(1)
C24-C23	1.35(1)	C24-C25	1.35(1)

Table S9/15. Bond angles [o] in **1**.

N21#1-Ag2-N21#2	115.9(2)	N21#1-Ag2-N21	97.3(3)
N21#2-Ag2-N21	115.9(2)	N21#1-Ag2-N21#3	115.9(2)
N21#2-Ag2-N21#3	97.3(3)	N21-Ag2-N21#3	115.9(2)
O5#4-Mn2-O5#5	108.0(3)	O5#4-Mn2-O5#6	108.0(3)
O5#5-Mn2-O5#6	112.4(7)	O5#4-Mn2-O5	112.4(7)
O5#5-Mn2-O5	108.0(3)	O5#6-Mn2-O5	108.0(3)
O2-Mn1-O3	110.9(4)	O2-Mn1-O1	111.8(7)
O3-Mn1-O1	107.1(4)	O2-Mn1-O4	110.0(4)
O3-Mn1-O4	109.7(3)	O1-Mn1-O4	107.2(4)
N11-Ag1-N1	174.0(2)	C2-N1-C6	117.2(6)
C2-N1-Ag1	122.4(5)	C6-N1-Ag1	120.3(5)
C16-N11-C12	116.9(6)	C16-N11-Ag1	121.1(5)
C12-N11-Ag1	122.0(5)	C13-C14-C15	119.5(7)
N1-C2-C3	122.7(7)	N1-C6-C5	122.6(7)
N11-C12-C13	123.5(7)	N11-C16-C15	122.9(8)
C4-C3-C2	118.7(8)	C14-C13-C12	118.1(8)
C16-C15-C14	119.1(8)	C5-C4-C3	119.3(7)
C4-C5-C6	119.4(8)	C22-N21-C26	116.1(8)
C22-N21-Ag2	119.7(6)	C26-N21-Ag2	124.2(6)
N21-C22-C23	124.9(8)	N21-C26-C25	122.4(8)
C23-C24-C25	119(1)	C22-C23-C24	118.4(9)
C24-C25-C26	118.9(9)		

Symmetry codes to generate equivalent atoms:
1. [2_755] -x+2,-y,z
2. [4_646] -y+1,x-*,-z+1
3. [3_666] y+1,-x+1,-z+1
4. [2_765] -x+2,-y+1,z
5. [7_564] y+1/2,-x+1/2-*,-z+1/2
6. [8_644] -y+1/2+1,x+1/2-*,-z+1/2

CCDC-1879263 contains the supplementary crystallographic data for this paper. These data can be obtained free of charge from The Cambridge Crystallographic Data Centre via www.ccdc.cam.ac.uk/data_request/cif.
